# An Overview and Recent Developments in the Analysis of Multistate Processes

**DOI:** 10.1002/sim.70493

**Published:** 2026-05-14

**Authors:** Malka Gorfine, Richard J. Cook, Per Kragh Andersen, Terry M. Therneau, Pierre Joly, Hein Putter, Maja Pohar Perme, Michal Abrahamowicz, Topic Group 8 “Survival Analysis” of the STRATOS Initiative

**Affiliations:** ^1^ Department of Statistics and Operations Research Tel Aviv University Tel Aviv Israel; ^2^ Department of Statistics and Actuarial Science University of Waterloo Waterloo Canada; ^3^ Section of Biostatistics University of Copenhagen Copenhagen Denmark; ^4^ Division of Biomedical Statistics and Informatics Mayo Clinic Rochester New York USA; ^5^ Inserm, ISPED, Bordeaux Populations Health Research Center University of Bordeaux Bordeaux France; ^6^ Department of Biomedical Data Sciences Leiden University Medical Center Leiden the Netherlands; ^7^ Department of Biostatistics and Medical Informatics, Medical Faculty University of Ljubljana Ljubljana Slovenia; ^8^ Department of Epidemiology, Biostatistics and Occupational Health McGill University Montreal Canada

**Keywords:** frailty, process history, pseudo‐values, state occupancy probability, time‐dependent covariates, transition intensity

## Abstract

Multistate models offer a powerful framework for studying disease processes and can be used to formulate intensity‐based and more descriptive marginal regression models. They also represent a natural foundation for the construction of joint models for disease processes and dynamic marker processes, as well as joint models incorporating random censoring and intermittent observation times. This article reviews the ways multistate models can be formed and fitted to life history data. Recent work on pseudo‐values and the incorporation of random effects to model dependence on the process history and between‐process heterogeneity are also discussed. The software available to facilitate such analyses is listed.

## 1 Introduction

### 1.1 Background

Long‐term cohort studies offer an excellent source of data for research on the onset and progression of chronic disease processes. When interest lies in the time to a particular event, insights are often gained from use of survival analysis methods. Yet, in many settings individuals may experience several types of events (e.g., myocardial infarctions, bleeds, non‐fatal stroke, and death in cardiovascular research) and interest may lie in studying the co‐occurrence of these events and the relationship between their event times. Multistate models offer a versatile framework for studying such processes through the analysis of transition rates between different health states. Our objectives are to provide a review of the general frameworks and methodology for multistate analysis, to discuss the formulation, estimation, and interpretation of alternative models, and to illustrate their application. The paper follows our first guidance paper on intensity‐based models for time to event analyses [1]. It is written primarily for readers familiar with concepts and notation of survival analysis which we generalize to accommodate more complex processes. We stress the connection between the multistate likelihood and partial likelihood used routinely in survival analysis, which facilitates use of modern software for survival analysis for the analysis of multistate processes. We hope this overview will promote widespread informed use of multistate models in research on human health and advance the general aims of the STRATOS Initiative [2].

The international STRATOS (STRengthening Analytical Thinking for Observational Studies) initiative aims to bridge the gap between recent advances in statistical methodology and the methods commonly used in applied observational research [2]. This gap largely stems from the lack of well‐documented guidance for analyzing observational data, unlike the widely adopted CONSORT guidelines for randomized clinical trials (RCTs) [3]. Observational studies often involve more diverse objectives, designs, and data structures than RCTs, posing complex analytical challenges that require a broad range of statistical methods. While methodological innovations continue to emerge, applied health research frequently relies on a narrow set of traditional approaches. To improve the validity of conclusions from observational studies, STRATOS develops evidence‐based guidance tailored to researchers with varying levels of statistical expertise. Initially, STRATOS comprised seven Topic Groups (TGs), each addressing a specific area of statistical analysis [2]. The Survival Analysis group (TG8) was added in 2015 to provide guidance on time‐to‐event data, with its first contribution published in 2021, focusing on intensity‐based modeling of failure time processes [1]. The present manuscript extends this work to the multistate setting.

Diseases involving distinct stages can be naturally characterized using multistate models. The states may represent different stages of a progressive condition such as hepatitis [4], the presence or absence of symptoms in episodic conditions such as chronic bronchitis [5], different phases of a response to treatment in cancer clinical trials [6, 7], or the course of a COVID‐19 infection as individuals move between moderate, severe, and critical disease states in hospital, become discharged, or die [8, 9]. In other settings, multistate models have been used to characterize the passage of insects through successive developmental stages of their life cycle [10], the formation and dissolution of marriages [11], or changes in the employment status of individuals in the workforce [12]. Careful modeling of process dynamics can yield valuable scientific insights regarding the natural disease course, risk factors for disease onset and progression, and intervention effects. Multistate models also offer a useful framework for the specification of joint models for time‐dependent covariate processes and disease processes of interest; such models can improve understanding of the dynamic relationship between two or more processes.

**FIGURE 1:**
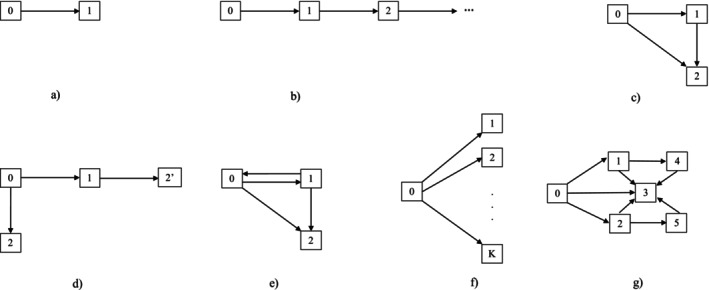
Some common processes represented as multistate models. (a) A 2‐state failure process. (b) A recurrent event process or progressive process. (c) A 3‐state illness‐death process. (d) A 4‐state illness‐death process. (e) A reversible illness‐death process. (f) A competing‐risks process. (g) An example of a more complex model.

The formation of multistate models begins with the specification of a set of states 𝒮 representing different conditions of the process. These typically correspond to different states of health or different stages of a disease process. For example, in degenerative conditions there are often grading systems to quantify the degree of damage—the modified Steinbrocker scoring system grades the degree of joint damage in rheumatology based on radiographic images [13], and the extent of liver damage in hepatitis C infection is graded according to the categories: fibrosis, fibrosis with portal expansion, bridging fibrosis, and cirrhosis [14]. States may also be defined by discretizing continuous markers—this is a strategy routinely adopted to facilitate modeling when processes are under intermittent observation; Satten and Longini [15], for example, study immune function in HIV by modeling change in states defined by ranges of CD4 cell counts in conjunction with the diagnosis of AIDS. In other settings, states are defined simply according to the occurrence of events. In cancer trials, for example, it is common to distinguish the states of being alive and recurrence‐free, alive following recurrence, and dead; the dead state can be further distinguished according to whether death occurred without or following recurrence. The set of states 𝒮 typically has a finite number of elements we label with integers {0,1,2,…,K}, say, but processes with a countable number of states can also be modeled (e.g., recurrent event processes) [16]. There is often a subset of states that can be entered directly from a given state with this subset determined from the context. Figure 1 contains some illustrative state‐space diagrams representing some of the rich variety of processes that can be analyzed using multistate models. The arrows in a state‐space diagram represent the transitions that can be made directly. When processes can terminate, the set of absorbing states from which individuals cannot exit is denoted by 𝒜.

Figure 1a is a two‐state diagram representing a simple failure process ideal for survival analysis with state 0 a transient state and state 1 an absorbing state entered upon failure (e.g., death). Figure 1b is a multistate diagram representing a recurrent event process [17] where the state labels correspond to the cumulative number of events experienced; there is no absorbing state represented here. Figure 1c is a conventional illness‐death model, which is useful for describing many processes in public health, such as disease onset or progression, where death may also need to be accommodated. For disease incidence, state 0 may represent an initial healthy state, state 1 is entered upon disease onset and state 2 is entered upon death which may happen in disease‐free individuals (as a 0→2 transition), or after the development of the disease (corresponding to a 1→2 transition). Figure 1d is an alternative representation of an illness death model with $𝒜=\{2,2^{\prime }\}$ where the different absorbing states convey whether deaths occurred in disease‐free or diseased individuals. This distinction between Figure 1c and d may seem trivial but the distinction between event‐free death and death following an intermediate event can enable more informative descriptive analyses. Figure 1e is a reversible illness‐death model which, because the 1→0 transition is allowed, would be suitable for modeling the course of chronic obstructive pulmonary disease (COPD) where exacerbation of symptoms may develop and resolve over time [18]. The setting of K+1 states with K competing events is depicted in Figure 1f. Much more elaborate state spaces can be defined to represent more complex processes. For example, Figure 1g represents a semi‐competing risk problem with multiple non‐fatal events and the principles and methods we discuss next apply in such settings. Recent applications in complex multistate disease settings includes models of COVID‐19 patient disease trajectories as transitions among five clinical states, mild or moderate, severe, critical, discharged, and deceased, while allowing for reversibility between selected states [8, 9]. Another example is the study of predictors of changes in employment status among individuals living with multiple sclerosis, which uses a three‐state multistate model comprising unemployment, part‐time employment, and full‐time employment, with reversible transitions permitted between all states [12].

In any multistate model, a time origin must be specified. This can sometimes be obvious as in the case where it is the birth of an individual, but sometimes its specification can be challenging. Discrete‐time models can be adopted but we focus on continuous‐time models in which event times take values on the positive real line.

### 1.2 Examples of Multistate Processes

The following publicly available datasets are used throughout the text to illustrate various approaches to multistate analysis. Codes are available in the Supporting Information file.

#### 1.2.1 Recurrence and Death in Colon Cancer

In a clinical trial conducted in the 1980s to investigate the use of levamisole and fluorouracil as adjuvant therapies for resected colon carcinoma [19], a total of 929 patients with stage C disease were randomly assigned to one of three groups: observation, levamisole alone, or levamisole combined with fluorouracil. Enrollment of patients was begun in March 1984, and was completed in October 1987. The time to cancer recurrence and survival time were considered key outcomes. We consider the illness‐death process of Figure 1d, where state 1 is entered upon cancer recurrence, state 2 represents recurrence‐free death, and state $2^{\prime }$ represents post‐recurrence death. State 0 corresponds to the initial state at the time of treatment assignment which we set as the time origin (t=0). The dataset is available in the R package survival [20]. The use of levamisole has no effect, so we combine observation and levamisole arms as the control and code treatment as 5FU+Lev (1) versus Control (0). A total of 468 individuals experienced recurrence and 452 individuals were observed to die; most death (414) occurred following recurrence. We revisit this example in Section 2.2 to illustrate the estimation of cumulative transition intensities and state occupancy probabilities over time. In Section 2.4, we illustrate the findings from intensity‐based regression modeling.

#### 1.2.2 Joint Damage in Psoriatic Arthritis

A second study we consider involves the study of joint damage in individuals with psoriatic arthritis, an autoimmune condition characterized by joint inflammation and damage along with skin involvement. The University of Toronto Psoriatic Arthritis Clinic began recruiting patients in 1977 and follows them over time to study the disease process in a clinical setting [21]. Clinical and radiological assessments are scheduled at regular intervals but the examination times vary considerably between patients and over time within patients. Here we consider study of the progression of joint damage in 305 individuals with psoriatic arthritis from an exported dataset [22] available in the msm R package [23]. There are 305 patients represented with a mean of 5.5 years of follow‐up (min = 0.1, max = 19.2) when we restrict attention to individuals with a minimum of 2 visits—the average number of visits is 2.6 (min = 2, max = 7). Four damage states are defined representing: mild, moderate, severe, and very severe impairment. Specifically, state 0 corresponds to 0 damaged joints, state 1 to 1‐4 damaged joints, state 2 to 5‐9 damaged joints, and state 3 to 10 or more damaged joints. This is a progressive process (i.e., transitions are irreversible) with transitions occurring in continuous time, but information on the number of damaged joints is only collected during periodic radiographic examinations, scheduled to take place every two years. The actual timing of assessments varies considerably across individuals, resulting in random visit times. Figure 4a illustrates the recruitment times and subsequent times of radiographic examination for five patients. In Section 4, we consider Figure 1b, with four states and two covariates—based on the number of effusions and the sedimentation rate—and model their effect on transitions to more advanced states of damage. An effusion is a swelling of tissue around a joint due to a build‐up of fluid which signals the disease is in an active phase; the count of the number of effusions is a measure of disease activity at the joint or patient level. Erythrocyte sedimentation rate is a blood marker reflecting the systemic level of inflammation.

#### 1.2.3 Rotterdam Tumor Bank Data

Data from the Rotterdam tumor bank, which includes 1546 breast cancer patients who had node‐positive disease and underwent a tumor removal surgery between the years 1978–1993, are available in the survival R package [20]. We take the date of surgery for tumor removal as the time of origin for Figure 1d; date of relapse, date of relapse‐free death, and date of post‐relapse death are the respective entry times to states 1, 2, and $2^{\prime }$. Prognostic baseline variables are age at surgery, menopausal status, tumor size, tumor grade, number of positive lymph nodes, levels of estrogen and progesterone receptors in the initial biopsy, hormonal therapy, and chemotherapy. Of the 1546 patients, 924 experienced a relapse of the disease (63%), 106 died without evidence of relapse (7%), and 771 patients died after a relapse (79% of the patients who showed a relapse of the cancer). This dataset is used to demonstrate the frailty‐based methods discussed in Section 5.1.

### 1.3 Overview of the Paper

In Section 2.1, we define intensity functions which serve as the building blocks for full models of multistate processes. Different classes of intensity functions are introduced, distinguished by the time scale governing risk. We then discuss computation of transition probability matrices for Markov processes and consider functionals of intensities, which are often the target of inference. Nonparametric estimation methods for a single sample with processes subject to right censoring, are presented in Section 2.2. In Section 2.3, we discuss the formulation of multiplicative intensity‐based models to study the effect of fixed or time‐varying covariates on transition rates. In Section 2.4, we derive the likelihood based on a joint model for time‐varying covariates, a censoring time, and the process of interest. Section 3 focuses on regression models aimed at estimating state occupancy probabilities using so‐called pseudo‐values. Section 4 addresses scenarios where processes operating in continuous time are observed intermittently, typically during clinic visits; see the example in Section 1.2.2. Section 5 explores the use of random effects in modeling multistate disease processes. Section 6 provides a review of statistical packages and functions available for multistate analysis. The advantages and limitations of multistate analysis are summarized in Section 7, while concluding remarks and directions for future research are presented in Section 8. For ease of reference, Section S5 of the Supporting Information file provides a glossary of the notation used throughout the paper.

## 2 Methodology

### 2.1 Notation and Foundations

We let t=0 represent the time origin of the processes under study and assume they are observed from their onset unless otherwise specified. Here we consider three types of notation with which multistate processes can be represented. We define this notation for the processes under study and discuss the notation for data on such processes in subsequent sections; see Section 2.2, for example. For progressive processes wherein each state can be entered at most once, it is sometimes convenient to define $T_{k}$ as the entry time to state k. If states are recurrent then one can let $T_{k}^{\left(r\right)}$ denote the rth entry time to state k, r=1,…, k=0,1,…. An issue with this representation is that entry times for some states do not exist (i.e., when the state is not entered) and when this is the case the associated distributions are improper. A compact alternative representation is to use Z(t)∈𝒮 for the state occupied at time t≥0 and {Z(s),0≤s} to represent the stochastic multistate process, where H(t)={Z(s),0≤s<t} is the history of the process—a record of the number, types, and times of transitions over [0,t). We assume Pr(Z(0)=0)=1 in what follows unless otherwise stated. Counting process notation yields a third powerful and convenient representation of multistate processes—here we let $N_{kl}(t)$ record the number of direct k→l transitions over [0,t]; $\left\{N_{kl}(t),0\leq s\right\}$ increments by one at $T_{l}^{\left(r\right)}$ if state l is entered from state k at $T_{l}^{\left(r\right)}$. Counting process notation is used in modern research on life history processes because it easily accommodates very general processes and aligns with the martingale representation and theory used to prove large sample properties of estimators [24].

Intensity functions are the fundamental building blocks of multistate processes, with the k→l intensity function given by

(1) $$\lambda_{kl}(t|H(t))=\underset{\Delta t↓0}{\lim}\frac{1}{\Delta t}\mathrm{Pr}\left(Z({\left(t+\Delta t\right)}^{-})=l|Z(t^{-})=k,H(t)\right)\;,\;\;\;k\neq l,$$

where $t^{-}$ denotes infinitesimal amount of time before t. This therefore represents the instantaneous risk per time unit of a k→l transition at time t given state k is occupied at $t^{-}$ and the history which records the number and types of all transitions over [0,t). The representation (1) is very general and modeling requires explicit specification of how the history affects the risk of transitions; while this is a powerful framework it can be a daunting challenge to specify the nature of this history dependence correctly but there are some special models that are useful for a wide range of applications.

For Markov processes, the k→l intensity function does not depend on the process history beyond the fact that state k is occupied at $t^{-}$ so we write $\lambda_{kl}(t|H(t))=\lambda_{kl}(t|Z(t^{-})=k)=\lambda_{kl}(t)$. In an early exploration of the use of Markov processes, Fix and Neyman [25] model cancer recurrence, death and loss to follow‐up with time‐homogeneous transition intensities for which $\lambda_{kl}(t)=\lambda_{kl}$ for (k,l)∈{(0,1),(0,2),(1,2)}. Time homogeneous models were used routinely in early work on multistate modeling and these—along with related weakly parametric models [26]—remain useful when processes are under intermittent observation. We point out in Section 2.2 that natural nonparametric estimates are easily obtained with right‐censored data and emphasize semiparametric methods in regression settings. For semi‐Markov processes, the intensity depends on the time since entry to state k, so we write $\lambda_{kl}(t|H(t))=\lambda_{kl}(B(t)|Z(t^{-})=k)$ where $B(t)=t-t_{k}^{\left(n_{k}(t)\right)}$ with $n_{k}(t)=\sum_{j\geq 0}N_{jk}(t^{-})$ the total number of times state k was entered over [0,t) and $t_{k}^{\left(n_{k}(t)\right)}$ the time that state k was most recently entered at t>0. Multistate processes may involve some intensities with a Markov form and others a semi‐Markov form, while others may involve hybrid time‐scales. For example, in chronic obstructive pulmonary disease, individuals may have recurrent exacerbations of symptoms, which may arise with increasing frequency with longer disease duration; the time to resolution of an exacerbation may also tend to increase with increasing disease duration. The intensity in such cases can involve specification of a basic time scale and incorporate dependence on other aspects of time via regression. For instance, one can adopt models of the form $\lambda_{kl}(t|H(t))=\lambda_{kl}\left(B(t)|Z(t^{-})=k\right)\exp\left(g{\left(t\right)}^{\text{T}}\gamma \right)$ where g(t) is a function of time and γ is a parameter that characterizes the dependence on t; that is a semi‐Markov model is obtained if γ=0.

The continuous‐time Markov model is a canonical model that warrants special attention. As noted earlier, the intensity $\lambda_{kl}(t|H(t))=\lambda_{kl}(t)$ for such processes, and we define the cumulative transition intensity as $\Lambda_{kl}(t)=\int_{0}^{t}d\Lambda_{kl}(s)$ where $d\Lambda_{kl}(s)=\lambda_{kl}(s)ds$. With state space 𝒮={0,1,…,K}, a (K+1)×(K+1) transition intensity matrix dΛ(s) can be formed with off‐diagonal entries $d\Lambda_{kl}(s)$ and (k,k) diagonal entry $-\sum_{l\neq k}d\Lambda_{kl}(s)$; the rows and columns are ordered here to correspond to the ordering of states 0,1,…,K, respectively. Consider a partition of the interval [s,t] defined by $s=u_{0}<u_{1}<\cdots <u_{R}=t$ with $\Delta u_{r}=u_{r}^{-}-u_{r-1}^{-}$ and define $\Delta \Lambda (u_{r})=\Lambda (u_{r}^{-})-\Lambda (u_{r-1}^{-})$, r=1,…,R. With I a (K+1)×(K+1) identity matrix, note that $I+\Delta \Lambda (u_{r})$ can be viewed as an approximation to a (K+1)×(K+1) transition probability matrix characterizing the state occupancy distribution at $u_{r}$ given state occupied at $u_{r-1}$; specifically the (k,l) entry (k≠l) of $I+\Delta \Lambda (u_{r})$ with $\Delta \Lambda_{kl}(u_{r})=\lambda_{kl}(u_{r-1})\Delta u_{r}$, approximates the probability that state l is occupied at $u_{r}$ given state k was occupied at $u_{r-1}$; the (k,k) diagonal entry $1-\Delta \Lambda_{k\cdot }(u_{r})$ approximates the probability that no transition is made given state k was occupied at $u_{r-1}$. Then in light of the partition, the product

$$\begin{array}{l} \overset{R}{\underset{r=1}{\prod }}\{I+\Delta \Lambda (u_{r})\} \end{array}$$

approximates the transition probability matrix P(s,t). This approximation improves with larger R values and if we take the limits, R→∞ and $\max(u_{r}-u_{r-1})\to 0$, we write

(2) $$P(s,t)=\underset{R↑\infty }{\lim}\;\overset{R}{\underset{r=1}{\prod }}\left\{I+\Delta \Lambda (u_{r})\right\}=\underset{\left(s,t\right]}{\prod }\left\{I+d\Lambda (u)\right\}$$

where the final term is simply the notation used to represent a product integral [24].

Having obtained a (K+1)×(K+1) transition probabilities matrix P(s,t) with (k,l) entry $p_{kl}(s,t)=\mathrm{Pr}(Z(t)=l|Z(s)=k)$, we are now in a position to use this matrix to describe features of the multistate process. When Z(0)=0 and s=0 in (2), $p_{k}(t)=\mathrm{Pr}(Z(t)=k|Z(0)=0)$ is the probability that state k is occupied at time t>0. This enables us to define a wide range of marginal features, including:

i. The probability that the process is in one of a set ${𝒮}^{†}$ of states is given by $\sum_{k\in {𝒮}^{†}}p_{k}(t)$, ${𝒮}^{†}\subseteq 𝒮$. If ${𝒮}^{†}=𝒜$ this corresponds to the probability that the process has terminated by time t>0, and if ${𝒮}^{†}\subset 𝒜$ then this is the probability that the process terminated due to absorption into a specific subset of the absorbing states.
ii. The expected total sojourn time spent in state k is $\mu_{k}=\int_{0}^{\infty }p_{k}(u)du$, where sojourn time is the amount of time spent in a particular state before moving to another state; we use the term “total sojourn time” for the time allowing for multiple spells in a given state. A *restricted mean sojourn time* over the interval (0,τ] can be defined by specifying a finite upper limit of integration, expressed as $\mu_{k}(\tau )=\int_{0}^{\tau }p_{k}(u)du$.
iii. For progressive processes, the cumulative incidence function for state k, given by $F_{k}(t)=\mathrm{Pr}(T_{k}\leq t)=\sum_{j\in {𝒮}_{k}}p_{j}(t)$ where ${𝒮}_{k}\subseteq 𝒮$ includes state k and any states that can be entered following a sojourn in state k. This is the probability of having entered state k by time t.

Here, the term *marginal* refers to functionals of the multistate process defined without conditioning on intermediate events or time‐varying covariates realized after the time origin. When data are subject to left truncation, direct estimation of marginal features may be more involved. As we discuss in Section 3.1, analyses should condition on the process history to support the assumption of independent delayed entry. With transition intensities estimated following such conditioning, marginal features can then be estimated by computation involving model assumptions. We next discuss the nonparametric estimation of P(s,t), which is relevant in many real‐world applications as a descriptive analysis, as illustrated in Section 2.2.

### 2.2 Censored Data, Nonparametric Estimation, and Descriptive Functionals

Although time‐homogeneous Markov processes assume constant transition intensities, this assumption can be relaxed in a straightforward manner by allowing the intensities to vary with time while retaining the Markov property. In particular, parametric models can be easily extended to accommodate temporal trends in transition risks; piecewise‐constant intensities provide a simple and practical formulation, yielding estimates of transition rates over prespecified time intervals and proving especially useful when processes are observed intermittently; see Section 4. Here we consider a one‐sample problem in which processes are under continuous observation from a common time origin t=0, but subject to right‐censoring, and for this setting nonparametric estimation is relatively straightforward. In what follows, we consider a single sample of n independent processes for individuals observed to a maximum time τ, a fixed administrative censoring time. We consider this as a planned fixed and common duration of follow‐up but it can, in principle, vary between individuals. To accommodate loss to follow‐up, let $C_{i}^{*}$ denote a random right‐censoring time so that individual i is observed continuously over the interval $\left[0,C_{i}\right]$ where $C_{i}=\min(\overset{*}{\underset{i}{C}},\tau )$. We assume here that $C_{i}^{*}$ is independent of the process {Z(s),0<s}, but comment on this more in Section 2.4.

Defining the at‐risk process and counting processes for events is more involved in a general multistate setting compared to the single failure time setting. The function $Y_{i}(t)=I(t\leq C_{i})$ indicates whether the process for individual i is under observation (i.e., is uncensored) at time t>0. If $N_{ikl}(t)$ is the total number of k→l transitions over [0,t] for process i, $\Delta N_{ikl}(t)=N_{ikl}({\left(t+\Delta t\right)}^{-})-N_{ikl}(t^{-})$ represents the number of k→l transitions for individual i over [t,t+Δt), and $dN_{ikl}(t)=\lim_{\Delta t↓0}\Delta N_{ikl}(t)$ is an indicator that they experienced a k→l transition at t. Let $Y_{ik}(t)=I(Z_{i}(t)=k)$ indicate that state k is occupied at t by individual i, and to distinguish the underlying counting process and the process observed under this censoring scheme, let ${\bar{Y}}_{ik}(t)=Y_{i}(t)Y_{ik}(t^{-})$ indicate a transition of individual i out of state k may be observed at time t. Then, let $d{\bar{N}}_{ikl}(t)={\bar{Y}}_{ik}(t)\;dN_{ikl}(t)$ indicate that a k→l transition is recorded for process i at time t, and ${\bar{N}}_{ikl}(t)=\int_{0}^{t}{\bar{Y}}_{ik}(s)\;dN_{ikl}(s)$ denote the total number of k→l transitions observed for process i over the interval [0,t]. If k∈𝒜, then $dN_{ikl}(t)$ is zero for all t>0 and all l∈𝒮, since absorbing states cannot be exited.

A natural estimator of $d\Lambda_{kl}(t)$ is given by

(3) $$d{\hat{\Lambda }}_{kl}(t)=\frac{d{\bar{N}}_{\cdot kl}(t)}{{\bar{Y}}_{\cdot k}(t)}$$

where $d{\bar{N}}_{\cdot kl}(t)=\sum_{i=1}^{n}{\bar{Y}}_{ik}(t)dN_{ikl}(t)$ is the total number of k→l transitions at time t observed in the sample, and ${\bar{Y}}_{\cdot k}(t)=\sum_{i=1}^{n}{\bar{Y}}_{ik}(t)$ is the total number of individuals at risk of a k→l transition in the sample at time t. This is analogous to the nonparametric estimate of the increment in the cumulative hazard at time t in survival analysis which also has the form “number of events in the sample at time t>0, divided by the size of the risk set at time t.” Note that we require ${\bar{Y}}_{\cdot k}(t)>0$ for this estimate to be defined at t and by convention we take $d{\hat{\Lambda }}_{kl}(t)=0$ when this is not satisfied. The Nelson‐Aalen estimator of $\Lambda_{kl}(t)$ is then

(4) $${\hat{\Lambda }}_{kl}(t)=\int_{0}^{t}d{\hat{\Lambda }}_{kl}(u),$$

which is a Stieltjes integral representation of a discrete sum of the distinct k→l transition times over (0,t] [24]. It is apparent from (3) that the integrand will be zero except at times when k→l transitions are observed.

The Aalen‐Johansen estimator [27] of the transition probability matrix P(s,t) is obtained by replacing the unknown quantities in the right‐hand side of (2) with the estimates given by (4), to obtain

(5) $$\hat{P}(s,t)=\underset{\left(s,t\right]}{\prod }\left\{I+d\hat{\Lambda }(u)\right\}$$

where $\hat{\Lambda }(u)$ is the matrix of estimated cumulative transition intensities obtained by replacing $\Lambda_{kl}(t)$ with the Nelson‐Aalen estimator.

If processes are observed from s=0 and Pr(Z(0)=0)=1, the top row of $\hat{P}(s,t)$ contains the Aalen‐Johansen estimator of the state k occupancy probability, $p_{k}(t)$, k=0,1,…,K. This in turn enables estimation of the functionals (i)—(iii) along with many others. Note in particular that if we have a two‐state survival process with states 0 and 1, the estimate (4) of $\Lambda_{01}(t)$ is the Nelson‐Aalen estimate of the cumulative hazard function and by applying (5) we obtain the Kaplan‐Meier estimate of the survival probability as ${\hat{p}}_{0}(t)$, the top left entry of the 2×2 matrix $\hat{P}(0,t)$.

Importantly, while the nonparametric estimator (5) is motivated by the Markov assumption, the estimates of $p_{k}(t)$ are robust and valid for non‐Markov processes provided censoring is completely independent of the multistate process [28, 29]. The infinitesimal jackknife offers a remarkably accurate approach to robust variance estimation in this setting; this is implemented in the survival library in R [7]. This means that the estimate (5) and inferences based on it can be valid for a broad range of processes [?].

#### 2.2.1 The Colon Cancer Study Revisited, I

To illustrate, we consider the colon cancer data set, introduced in Section 1.2.1. The code is available in Section S1 of the Supporting Information file. The data frame is structured in the “counting process” format, making it suitable for analyzing in terms of a broad class of multistate processes. In this format, the follow‐up period for each individual is divided into intervals during which the individual is at risk of transitioning from one state to any other possible state. The presence of the ${\bar{Y}}_{ik}(t)$ term in the denominator of Equation (3) necessitates tracking when individuals occupy different states, specifically when they are at risk of transitioning out of state k.

**FIGURE 2:**
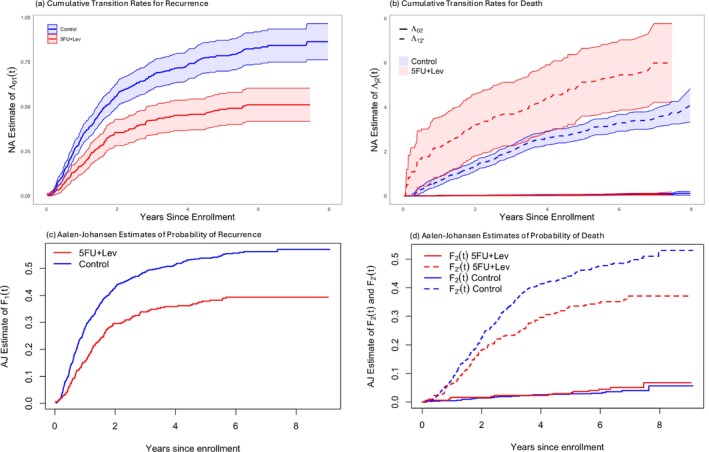
Colon cancer study: (a) Nelson‐Aalen (NA) estimates of cumulative transition intensity for recurrence (0→1) along with a pointwise 95% confidence interval. (b) NA estimates of cumulative transition intensity into death state (0→2 and $0\to 2^{\prime }$) along with a pointwise 95% confidence interval. (c) Aalen‐Johansen (AJ) estimates of the cumulative incidence functions of recurrence. (d) AJ estimates of the cumulative incidence functions of recurrence‐free death and post‐recurrence death.

Figure 2a shows the Nelson‐Aalen estimates of the cumulative transition intensity for recurrence (0→1) along with pointwise 95% confidence intervals. The slope of these estimates convey how the recurrence intensity (i.e., risk of recurrence among individuals who are alive and recurrence‐free) changes over time. The estimate for the control group shows a roughly constant intensity over the first two years, followed by a period with a lower intensity leading to decreased slope. The Nelson‐Aalen estimate for the 5FU+Lev group has a lower initial intensity over the first two years, which also levels off afterward. Figure 2b presents the Nelson‐Aalen estimates for transitions into death states (0→2 and $1\to 2^{\prime }$) and pointwise 95% confidence intervals. The very small estimates reflect that relatively few individuals make transitions directly from state 0 to 2 for either treatment group. Among individuals who experience early recurrence, however, those individuals receiving 5FU+Lev have an elevated intensity for death; see the sharp increase within the first six months. There is no sharp increase in the cumulative intensities for the control group with the estimate for the 0→2 transition very small for the range of time considered. However, for state k=1 the denominator of Equation (3) may be very small in the first few months and this may be an artefact—the slopes of $1\to 2^{\prime }$ are roughly similar after this initial six months period. Figure 2b suggests a slightly elevated risk of death in the 5FU+Lev group compared to the control arm. The overall reduction in post‐recurrence mortality in the 5FU+Lev group is primarily due to a lower risk of recurrence. The separation in the Nelson‐Aalen estimates is largely driven by differences observed during the early follow‐up period, when the number of subjects at risk is relatively small.

If $T_{1}$ is the time of entry into state 1, then the cumulative incidence function for recurrence, $F_{1}(t)=\mathrm{Pr}(T_{1}\leq t)$, can be expressed as the probability of having entered state 1 by time t, given by $F_{1}(t)=p_{1}(t)+p_{2^{\prime }}(t)$. Likewise, the cumulative incidence function for recurrence‐free death is defined as $F_{2}(t)=p_{2}(t)$, while the probability of death following recurrence is given by $F_{2^{\prime }}(t)=p_{2^{\prime }}(t)$. Note that these are all sub‐distribution functions because they do not approach 1 as t→∞ due to the presence of competing risks. Such functions, however, have the appealing feature of being interpretable as probabilities.

The Aalen‐Johansen estimates of $F_{1}(t)$ are shown in Figure 2c for each treatment group. It is evident that the 5FU+Lev group has an appreciably lower risk of recurrence. Figure 2d presents the estimates for the cumulative incidence functions of death‐related events. Evidently, the risk of recurrence‐free death is low in both treatment groups, and 5FU+Lev is associated with a reduced risk of post‐recurrence death.

### 2.3 Intensity‐Based Regression Models

If interest lies in assessing the relationship between time‐varying covariates and the multistate process, this can be studied through intensity‐based regression models [16, 24, 30, 31]. Let X(t) represent a p×1 covariate at t and {X(s),0≤s} denote the covariate process. If ℋ(t)={Z(s),X(s),0≤s<t} is the expanded history including information on the covariate path, the intensity function can be modified by replacing H(t) with ℋ(t) in Equation (1). Intensity‐based regression models aim to characterize how the instantaneous risk of a k→l transition depends on the features of the covariate process. Time‐dependent covariates can represent external factors such as season, air pollution, and so forth. or may be based on auxiliary features of the disease process; often interest lies in examining how marker processes may relate to the disease process of interest. Cholesterol levels, for example, may be recorded in cardiovascular trials and interest may lie in relating these to the risks of cardiac events, hospitalization, or death. One may also define time‐dependent covariates to summarize important aspects of the process history to model the history dependence via regression. The most common form is the multiplicative model [16, 24, 31, 32], where

(6) $$\lambda_{kl}(t|ℋ(t))=\lambda_{kl}(t|H(t))\exp(X{\left(t\right)}^{\text{T}}\beta_{kl}).$$

The vector of coefficients $\beta_{kl}$ characterizes the effect of covariates on the k→l transition intensity. Specifically, $\exp(\beta_{kls})$ is the relative risk of a k→l transition associated with a one unit increase in the sth covariate of X(t), whereas all other covariate values are unchanged. In survival analysis, the term hazard ratio is often used but relative risk is a broader concept that is more suitable in intensity‐based regression analysis. If $\lambda_{kl}(t|H(t))=\lambda_{kl}(t)$, this is called a modulated Markov model, where the covariate process modulates the baseline Markov intensity. This simple model can be used to examine the association between events; for example for an illness death process if $\lambda_{12}(t)$ differs from $\lambda_{02}(t)$ then the intermediate event impacts the risk of death. This can be studied by fitting models with the constraint $\lambda_{12}(t)=\lambda_{02}(t)\exp(\beta )$. Likewise, if $\lambda_{kl}(t|H(t))=\lambda_{kl}(B(t))$ in (6), this corresponds to a modulated semi‐Markov model [16]. When covariates are time fixed, conditional on the covariates X, these models reduce to Markov and semi‐Markov models, respectively. For Markov processes given covariates X, a (K+1)×(K+1) transition probability matrix P(s,t|X) can be defined with (k,l) entry Pr(Z(t)=l|Z(s)=k,X). This can be estimated by stratification if the covariates are discrete, or by fitting regression models like (6) and applying product integration as in (2). Covariate effects can also be expressed as having additive effects on the process intensities [28]. For non‐Markov processes, computing transition probabilities is more challenging [33, 34].

Conditional on the covariate process, the stochastic nature of the multistate process is fully specified by the set of transition intensities [24, 30]. When covariates are time‐varying, joint models for the covariate and disease processes are often useful. We next discuss constructing likelihoods in the presence of time‐dependent covariates and random censoring, and address likelihood construction under intermittent observation in Section 4.

### 2.4 Likelihood for Intensity‐Based Models With Time‐Dependent Covariates

When processes involve time‐dependent covariates and loss of follow‐up, it is important to recognize that these are random processes which play a role in the data generation. Here, we discuss likelihood construction with this in mind [16].

Let $C_{i}^{*}(t)=I(C_{i}^{*}\leq t)$ be the indicator of whether random censoring (e.g., loss of follow‐up) occurred by time t, and denote the corresponding counting process as $\left\{C_{i}^{*}(s),0\leq s\right\}$. Information about time‐varying covariates usually ceases when the multistate process enters an absorbing state or the process is censored and we assume this in what follows. Hence, the at‐risk process should be adjusted accordingly. Let $Y_{i}^{†}(t)=I(Z_{i}(t)\notin 𝒜)$ indicate that the occupied state at time t by individual i is a non‐absorbing state, ${\bar{Y}}_{i}(t)=Y_{i}(t)Y_{i}^{†}(t^{-})$ equals 1 if individual i may be observed to transit at time t. The vector ${\bar{N}}_{ik}(t)={\left({\bar{N}}_{ikl}(t),l\neq k,l=1,\ldots ,K\right)}^{\text{T}}$ records the cumulative number of transitions from state k over (0,t] where it is understood that these counts will be zero for state l that cannot be entered directly from state k. Finally, ${\bar{N}}_{i}(t)={\left({\bar{N}}_{ik}^{\text{T}}(t),k\notin 𝒜\right)}^{\text{T}}$ is the vector of all counting processes of non‐absorbing states. We define $\Delta {\bar{X}}_{i}(t)={\bar{Y}}_{i}(t+\Delta t)\{X_{i}({\left(t+\Delta t\right)}^{-})-X_{i}(t^{-})\}$ to represent an increment in the observed covariate vector over [t,t+Δt). Here, ${\bar{Y}}_{i}(t+\Delta t)$ ensures that the multistate process has not yet reached an absorbing state or been censored by t+Δt so the covariate can be observed. Additionally, $d{\bar{X}}_{i}(t)=\lim_{\Delta t↓0}\Delta {\bar{X}}_{i}(t)$ and ${\bar{X}}_{i}(t)=\int_{0}^{t}d{\bar{X}}_{i}(s)$. The history of the observed multistate, covariate, and random censoring processes is then denoted by ${\bar{ℋ}}_{i}(t)=\{Y_{i}(s),{\bar{N}}_{i}(s),{\bar{X}}_{i}(s),0\leq s<t;Z_{i}(0),X_{i}(0)\}$. The intensity for random censoring is then defined generally as

(7) $$\underset{\Delta t↓0}{\lim}\frac{1}{\Delta t}\mathrm{Pr}(\Delta \overset{*}{\underset{i}{C}}(t)=1|{\bar{ℋ}}_{i}(t))={\bar{Y}}_{i}(t)\;\lambda^{c}(t|{\bar{ℋ}}_{i}(t)),$$

where $\Delta C_{i}^{*}(t)=C_{i}^{*}({\left(t+\Delta t\right)}^{-})-C_{i}^{*}(t^{-})$. The term ${\bar{Y}}_{i}(t)$ in Equation (7) ensures that the censoring intensity is zero once the multistate process is censored or reaches an absorbing state.

To construct the full likelihood, we consider a partition of [0,τ] defined by the points $0=u_{0}<u_{1}<\cdots <u_{R}=\tau$. We then consider the contributions over the sub‐intervals $\left[u_{r-1},u_{r}\right)$, r=1,…,R. To this end we let $\Delta {\bar{X}}_{i}(u_{r})={\bar{Y}}_{i}(u_{r})\{X_{i}(u_{r})-X_{i}(u_{r-1})\}$ represent an increment in the covariate vector and let $\Delta {\bar{N}}_{i}(u_{r})=Y_{i}(u_{r})\{N_{i}(u_{r}^{-})-N_{i}(u_{r-1}^{-})\}$ denote the number of transitions over $\left[u_{r-1},u_{r}\right)$. Finally, ${\bar{H}}_{i}(u_{r})=\{Y_{i}(u_{s}),\Delta {\bar{N}}_{i}(u_{s}),\Delta {\bar{X}}_{i}(u_{s}),s=1,\ldots ,r;Z_{i}(0),X_{i}(0)\}$ is the history of the censoring and joint multistate and covariates processes over the partition. For interval $\left[u_{r-1},u_{r}\right)$, the following contribution is made by processes i:

$$\begin{array}{ll} & \left\{\left[\mathrm{Pr}\left(\Delta {\bar{X}}_{i}(u_{r})|\Delta {\bar{N}}_{i}(u_{r}),Y_{i}(u_{r})=1,{\bar{H}}_{i}(u_{r-1})\right)\;\right.\right.\\ & \;\mathrm{Pr}{\left.\left(\Delta {\bar{N}}_{i}(u_{r})|Y_{i}(u_{r})=1,{\bar{H}}_{i}(u_{r-1})\right)\right]}^{Y_{i}(u_{r})}\\ & \;\times \;\mathrm{Pr}{\left(\Delta \overset{*}{\underset{i}{C}}(u_{r})=1|{\bar{H}}_{i}(u_{r-1})\right)}^{\Delta C_{i}^{*}(u_{r})}\\ & \;\mathrm{Pr}{\left.{\left(\Delta C_{i}^{*}(u_{r})=0|{\bar{H}}_{i}(u_{r-1})\right)}^{1-\Delta C_{i}^{*}(u_{r})}\right\}}^{Y_{i}(u_{r-1})I(Z_{i}(u_{r-1})\notin 𝒜)}\;. \end{array}$$

Note that a likelihood contribution is made over $\left[u_{r-1},u_{r}\right)$ by an individual only if they have not been censored and the multistate process is not in an absorbing state at time $u_{r-1}$. Second, there is a contribution related to the multistate and covariate processes only if the individual is not censored by time $u_{r}$. Third, by adopting the particular factorization here, the stochastic model for the increment in the covariate process is conditional not only on $\Delta {\bar{X}}_{i}(u_{r-1}),\ldots ,\Delta {\bar{X}}_{i}(u_{1}),X_{i}(0)$ but also on $\Delta {\bar{N}}_{i}(u_{r}),\ldots ,\Delta {\bar{N}}_{i}(u_{1})$, $Z_{i}(0)$ and $I(C_{i}>u_{r})$. This accommodates the setting in which covariates may cease to be defined when certain (usually absorbing) states are reached in the multistate process. This formulation requires covariates to be available in continuous time. This can narrow the scope of problems that can be handled, but in many applications covariates are constant between observable change‐points. Examples include settings where they record whether particular events have occurred or not. When discrete time‐dependent covariates are measured only at intermittent assessment times a joint multistate model can be formed while continuous time‐dependent covariates may lead to use of joint modeling techniques [35].

Under the partition $0=u_{0}<u_{1}<\cdots <u_{R}=\tau$, the full likelihood based on data of individual i over [0,τ] is the product of the following three terms:

(8) $$\overset{R}{\underset{r=1}{\prod }}\mathrm{Pr}{\left(\Delta {\bar{X}}_{i}(u_{r})|\Delta {\bar{N}}_{i}(u_{r}),Y_{i}(u_{r})=1,{\bar{H}}_{i}(u_{r-1})\right)}^{Y_{i}(u_{r})I(Z_{i}(u_{r-1})\notin 𝒜)}$$

pertaining to the covariate process,

(9) $$\overset{R}{\underset{r=1}{\prod }}\mathrm{Pr}{\left(\Delta {\bar{N}}_{i}(u_{r})|Y_{i}(u_{r})=1,{\bar{H}}_{i}(u_{r-1})\right)}^{Y_{i}(u_{r})I(Z_{i}(u_{r-1})\notin 𝒜)}$$

pertaining to the multistate process, and

$$\overset{R}{\underset{r=1}{\prod }}\mathrm{Pr}{\left(\Delta \overset{*}{\underset{i}{C}}(u_{r})|{\bar{H}}_{i}(u_{r-1})\right)}^{Y_{i}(u_{r-1})I(Z_{i}(u_{r-1})\notin 𝒜)}$$

for the random censoring process.

The censoring and covariate processes are said to be *noninformative* if there is no information to be gained about the parameters of primary interest (i.e., those indexing the multistate process) by modeling the censoring or covariate processes. Thus unless interest lies in joint modeling of a covariate (often termed a “marker process”) and the multistate process, under the assumption that the censoring and covariate processes are noninformative, it is customary to restrict attention to (9). This requires specification of intensity function for the observable counting process. We write the probability of a contribution for a particular interval $\left[u_{r-1},u_{r}\right)$ in (9) as

$$\begin{array}{ll} & \mathrm{Pr}\left(\Delta {\bar{N}}_{i}(u_{r})|Y_{i}(u_{r})=1,{\bar{H}}_{i}(u_{r-1})\right)\\ & \;=\overset{K}{\underset{k=1}{\prod }}\mathrm{Pr}{\left(\Delta {\bar{N}}_{ik}(u_{r})|Y_{i}(u_{r})=1,{\bar{H}}_{i}(u_{r-1})\right)}^{Y_{ik}(u_{r-1})}\;, \end{array}$$

which can be written more explicitly as

(10) $$\begin{array}{ll} & \overset{K}{\underset{k=1}{\prod }}\left\{\overset{K}{\underset{l\neq k=1}{\prod }}\mathrm{Pr}{\left(\Delta {\bar{N}}_{ikl}(u_{r})=1|Y_{i}(u_{r})=1,{\bar{H}}_{i}(u_{r-1})\right)}^{\Delta {\bar{N}}_{ikl}(u_{r})}\right.\\ & \;\mathrm{Pr}{\left.{\left(\Delta {\bar{N}}_{ik\cdot }(u_{r})=0|Y_{i}(u_{r})=1,{\bar{H}}_{i}(u_{r-1})\right)}^{1-\Delta {\bar{N}}_{ik\cdot }(u_{r})}\right\}}^{Y_{ik}(u_{r-1})}\;. \end{array}$$

where ${\bar{N}}_{ik.}(t)=\sum_{l\neq k=1}^{K}{\bar{N}}_{ikl}(t)$. To proceed further, it is necessary to define the intensity for the observable counting process

(11) $$\underset{\Delta t↓0}{\lim}\;\frac{1}{\Delta t}\mathrm{Pr}\left(\Delta {\bar{N}}_{ikl}(t)=1|Y_{i}(t)=1,{\bar{ℋ}}_{i}(t)\right).$$

To express this in terms of the intensities of the process of interest, we require an additional assumption that the random censoring is conditionally independent of the multistate process, given the history ${\bar{ℋ}}_{i}(\cdot )$ [24, 30, 36]. This is often simply referred to as independent censoring. Under this assumption the probability in the numerator of (11) is $\mathrm{Pr}(\Delta N_{ikl}(t)=1|{ℋ}_{i}(t))$, and we can write the intensity (11) as ${\bar{Y}}_{ik}(t)\;\lambda_{kl}(t|{ℋ}_{i}(t))$. Then, by expressing (11) in terms of $\lambda_{kl}(t|{ℋ}_{i}(t))$ and taking the limit as R→∞ we obtain

(12) $$L_{i}\propto \overset{K}{\underset{k=1}{\prod }}\overset{K}{\underset{l\neq k=1}{\prod }}L_{ikl}$$

where

(13) $$\begin{array}{ll} L_{ikl} & \propto \left\{\underset{t_{r}\in {𝒟}_{ikl}}{\prod }\lambda_{kl}\left(t_{r}|{ℋ}_{i}(t_{r})\right)\right\}\exp\left(-\int_{0}^{\infty }{\bar{Y}}_{ik}(u)\;\lambda_{kl}\left(u|{ℋ}_{i}(u)\right)\;du\right)\;, \end{array}$$

with ${𝒟}_{ikl}$ being the set of k→l transition times observed over [0,τ] for observation i. The likelihood contribution presented here corresponds to individual i. For a sample of n independent processes, the overall likelihood is the product of these terms, $\prod_{i=1}^{n}L_{i}$. If the elements $L_{ikl}$ do not share any parameters then optimization of (12) can be carried out by separately optimizing (13) which in turn can be carried out using standard software for survival analysis provided it can deal with left‐truncated and right‐censored data. The large sample properties of the resulting estimators follow immediately from those of standard survival analysis. See Andersen et al. [24] for the technical details and Aalen et al. [30], Cook and Lawless [16], and Andersen and Ravn [37] for related material.

#### 2.4.1 The Colon Cancer Study Revisited, II

**FIGURE 3:**
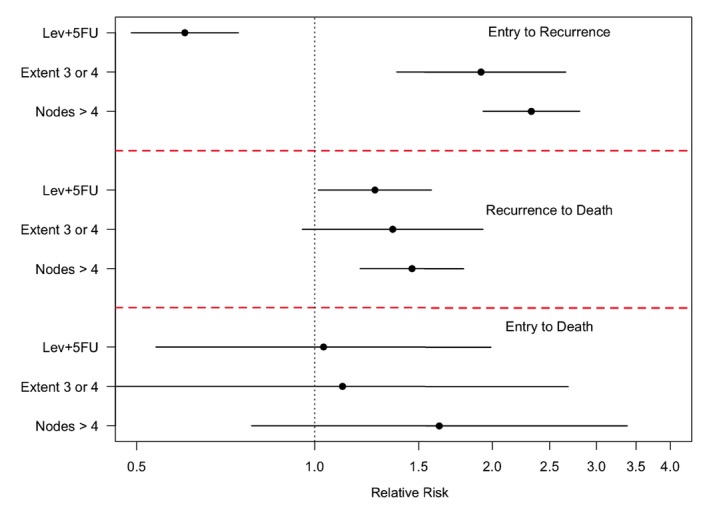
Colon cancer study: relative risk and the corresponding 95% confidence intervals from intensity‐based Cox regression analyses.

To illustrate the intensity‐based regression modeling, we examine three risk factors in the colon cancer study: treatment group (5FU+Lev versus control, denoted $X_{1}$); extent of invasion, defined as a binary variable with submucosa or muscle (values 1 or 2 in the dataset) versus serosa or contiguous structures (3 or 4) ($X_{2}$); and an indicator of more than 4 lymph nodes being involved ($X_{3}$). The actual number of lymph nodes involved may be a better reflection of disease stage but we dichotomize in this illustration. We then apply intensity‐based regression models of the form

$$\begin{array}{l} \lambda_{kl}(t|X_{i})=\lambda_{kl}(t)\exp\left(X_{i}^{\text{T}}\beta_{kl}\right) \end{array}$$

where $X_{i}={\left(X_{i1},X_{i2},X_{i3}\right)}^{\text{T}}$ and $\beta_{kl}$ is a vector of regression coefficients conveying the effect of covariates on the k→l intensity, $\left(k,l)\in \{(0,1),(0,2),(1,2^{\prime })\right\}$; see Figure 1d. Here, the Markov assumption implies that, conditional on the current state and covariates, transition intensities do not depend on the earlier process history. While convenient for modeling, this assumption may not be realistic in many applications. It can be seen in the code, available in Section S2 of the Supporting Information, that model‐based standard errors are reported; similar results are obtained when robust standard errors are specified. The results of Table 1 and Figure 3 show that the 5FU+Lev treatment significantly reduces the rate of recurrence when controlling for the extent of disease involvement and nodal involvement. Likewise, individuals with more extensive disease and those with more than 4 lymph nodes involved have significantly higher rate of recurrence when controlling for treatment. For recurrence‐free death, there is no evidence of an effect of any of the risk factors. For death following recurrence, one may be tempted to conclude there is possible harm from the treatment when controlling for the extent of disease and nodal involvement. However, we caution against such interpretation, since more comprehensive treatment of possible time‐dependent confounding factors is warranted. A more nuanced analysis of this process is warranted and would be possible with a larger set of possibly time‐dependent covariates. In this case, the main aim would be to investigate whether different types of individuals experience recurrence in the two arms, and, if so, to account for these differences when assessing the effect of treatment on post‐recurrence mortality. Alternatively, one may base analyses on pseudo‐values as we discuss in Section 3.4.

**TABLE 1:** Colon cancer study: Cox‐regression coefficients (Est), standard errors (SE) and relative risk (RR) from intensity‐based analyses.

Transition	Covariate	Est	SE	RR	p
Entry to recurrence, 0→1	5FU+Lev	−0.508	0.106	0.603	<0.001
	Extent 3 or 4	0.649	0.168	1.914	<0.001
	Nodes >4	0.845	0.096	2.328	<0.001
Recurrence to death, $1\to 2^{\prime }$	5FU+Lev	0.235	0.113	1.265	0.037
	Extent 3 or 4	0.304	0.179	1.355	0.091
	Nodes >4	0.379	0.103	1.461	<0.001
Entry to death, 0→2	5FU+Lev	0.031	0.333	1.035	0.917
	Extent 3 or 4	0.108	0.449	1.115	0.809
	Nodes >4	0.486	0.373	1.627	0.193

## 3 Other Modeling Considerations

### 3.1 Delayed Entry and Incomplete Data on Process History

The previous section covered intensity‐based modeling in an idealized scenario where individuals are observed from the onset of their process. While this is typical in inception cohorts, in many studies individuals are enrolled after the process has already been underway for some time. Recruitment is often a two‐step process: first, identifying individuals eligible for inclusion; second, obtaining their consent to participate. We initially assume that information on the pre‐selection history is available.

Let $A_{i}$ denote the recruitment time of individual i, after which we intend to observe their process over $\left(A_{i},\tau \right]$, say. In some settings, such as the UK Biobank [38, 39], information on certain transitions over $\left(0,A_{i}\right]$ is available (e.g., cancer diagnosis), whereas other events may be unrecorded (e.g., first diagnosis of hypertension). Let $Y_{i}^{A}(t)=I(A_{i}\leq t)$ and ${\tilde{Y}}_{i}(t)=Y_{i}^{A}(t)Y_{i}(t)Y_{i}^{†}(t^{-})$ indicate that individual i is under study (i.e., has been recruited and has not yet been censored or entered an absorbing state), and ${\tilde{Y}}_{ik}(t)=Y_{i}^{A}(t)Y_{i}(t)I(Z_{i}(t^{-})=k)$ indicates that the individual is under study and at risk for transition out of state k at time t. Note that we use $t^{-}$ in $I(Z_{i}(t^{-})=k)$ since they must be at risk at time t but use t for $Y_{i}^{A}(t)$ and $Y_{i}(t)$ since they must be under observation at t if we are to see any such transition. Similar to previous notation, let $d{Ñ}_{ikl}(t)={\tilde{Y}}_{ik}(t)dN_{ikl}(t)$, ${Ñ}_{ik}(t)={\left({Ñ}_{ikl}(t),l\neq k,l=1,\ldots ,K\right)}^{\text{T}}$ and ${Ñ}_{i}(t)={\left({Ñ}_{ik}^{\text{T}}(t),\notin 𝒜\right)}^{\text{T}}$. With time‐fixed covariates, the broadened history including the information on the delayed‐entry time may be written as ${\tilde{ℋ}}_{i}(t)=\{Y_{i}^{A}(s),Y_{i}(s),d{Ñ}_{i}(s),0\leq s<t,X_{i}\}$. This assumes that information on the process before $A_{i}$ is available, enabling the modeling of the intensity function over $\left(A_{i},\tau \right]$.

Under conditionally independent delayed entry [40] and conditionally independent loss to follow‐up

$$\underset{\Delta t↓0}{\lim}\;\frac{1}{\Delta t}\mathrm{Pr}\left(\Delta {Ñ}_{ikl}(t)=1|Z_{i}(t^{-})=k,{\tilde{ℋ}}_{i}(t)\right)={\tilde{Y}}_{ik}(t)\lambda_{kl}\left(t|{ℋ}_{i}(t)\right)\;.$$

The likelihood can then be constructed as in ((12), (13)), but with ${\bar{Y}}_{ik}(t)$ replaced by ${\tilde{Y}}_{ik}(t)$ and ${𝒟}_{ikl}$ being the set of transition times observed over $\left[A_{i},\tau \right)$. When censoring is conditionally independent but this equality does not hold, there is evidence of dependent delayed entry. Addressing dependent delayed entry is more challenging than addressing dependent loss of follow‐up since information on unselected individuals may be unknown. Acquisition of a representative sample or population data can both help gauge the extent of any bias and offer an avenue for mitigating the effect of selection bias [39, 41].

In settings where information on the process for $t<A_{i}$ is either completely missing or highly coarsened [42], fitting intensity‐based models that heavily depend on the process history becomes challenging. Efforts to obtain this history are warranted, as otherwise stronger simplifying modeling assumptions would be required. Although Markov models should be justified based on scientific plausibility and evidence of adequacy, they are particularly appealing for use in such situations, as the intensities are independent of the histories given the current state.

### 3.2 Time‐Dependent Covariates and Joint Modeling

The factorization of the likelihood given in Section 2.4 justifies the use of the likelihood based solely on the multistate process. However, joint modeling of covariates and multistate processes is of scientific value in settings where the interest lies in the relationship between the two processes. For example, when studying the role of markers of bone health in relation to the risk of fractures, continuous markers of bone formation and resorption could be incorporated into the intensities for the occurrence of first and subsequent bone fractures. Fractures, in turn, can affect bone markers, and this effect can be examined within a joint model for the two processes [43]. In this case, a likelihood based on (8) and (9) can be considered. If the continuous bone markers are discretized, a joint multistate model can be constructed, with states defined by combinations of marker levels and fracture states, potentially including an absorbing state for death. In this example, an additional challenge arises when covariates are subject to intermittent observation. We discuss how this can be addressed in Section 4.

### 3.3 Inference for Marginal Parameters and Pseudo‐Values

The intensity functions are the fundamental components of a multistate process, and, as shown above, specifying all intensity functions enables the construction of the likelihood. This further implies that all probabilistic aspects of the process are determined—at least when the intensity model does not include time‐dependent covariates that introduce ‘extra randomness’ beyond the multistate process itself (i.e., when the likelihood based on Equation (8) is straightforward). Thus, marginal features, such as state occupancy probabilities, $p_{k}(t)$, and expected sojourn times, $\mu_{k}$, in the states, can be estimated based on the estimated intensity functions, either through a “plug‐in” approach (if the mathematical relationship can be specified) or via simulation.

However, in a regression setting, the plug‐in approach does not provide parameters that directly describe the association between time‐fixed covariates, X and, for example, $p_{k}(t)$. Additionally, if the primary scientific interest lies in such an association, then typically *all* intensity functions need to be modeled, and model misspecification becomes a concern. It is therefore of interest to directly specify a marginal model for the association, that is, without relying on models for the intensity functions.

In general, it is not possible to specify intensity functions for the multistate process in such a way that a simple marginal model, such as (14), holds. Therefore, a direct marginal model should be seen as a ‘working model’, useful for assessing a direct association between the marginal parameter and X, but not necessarily reflecting the true data‐generating mechanism. For any regression model, simplifying assumptions, such as additivity and linearity, should be carefully evaluated through appropriate diagnostics.

Here, we discuss two recent approaches to direct marginal modeling: *direct binomial regression* using inverse probability of censoring weighted (IPCW) generalized estimating equations (GEE) [44, 45] and the *pseudo‐values* (PV) method [46, 47]; see also the recent book by Andersen and Ravn [37]. We illustrate these approaches by studying $p_{k}(t_{0})$ for a fixed time point $t_{0}$, but emphasize that similar methods can be applied for joint inference at multiple time points, $p_{k}(t_{0}),\ldots ,p_{k}(t_{m})$, or for the τ−restricted mean sojourn time in state k, $\mu_{k}(\tau )=\int_{0}^{\tau }p_{k}(u)du$. Additionally, conditional probabilities, such as $p_{kl}(s,t|X)=\mathrm{Pr}(Z(t)=l|Z(s)=k,X)$ or $p_{kl}(s,t|X(s))=\mathrm{Pr}(Z(t)=l|Z(s)=k,X(s))$ can be studied using these approaches via the method of *landmarking* [48, 49, 50].

Consider a regression model

(14) $$g\left(p_{k}(t_{0}|X_{i})\right)=X_{i}^{\text{T}}\beta$$

where $p_{k}(t|X_{i})=\mathrm{Pr}(Z_{i}(t)=k|Z_{i}(0)=0,X_{i}(0)=X_{i})$, g is a specified link function, and the coefficient vector β includes an intercept specific to the time point $t_{0}$. Thus, the coefficients will be specific to both the state, k, and the time point, $t_{0}$, though for ease of notation we denote coefficient vector as β. Typical link functions include the cloglog, corresponding to a proportional hazards model in the two‐state model (Figure 1a), or the logit function.

Direct binomial regression builds on those subjects for whom the state k indicator $I(Z_{i}(t_{0})=k)$ at time $t_{0}$ is observed. These are the subjects with $t_{0}\wedge T_{i}^{†}\leq C_{i}^{*}$, that is, either $t_{0}$ or $T_{i}^{†}=\inf_{t}\{Z_{i}(t)\in 𝒜\}$ (the time at which $Z_{i}(t)$ reaches an absorbing state) must occur before the time $C_{i}^{*}$ of random censoring. The state indicators $I(Z_{i}(t_{0})=k)$ for these subjects are then used as responses in a GEE, $U_{D}(\beta )=0$, with

(15) $$\begin{array}{ll} U_{D}(\beta ) & =\overset{n}{\underset{i=1}{\sum }}I\left(t_{0}\wedge T_{i}^{†}\leq C_{i}^{*}\right)W_{i}\left(t_{0}\wedge T_{i}^{†}\right)A\left(\beta ,X_{i}\right)\\ & \;\times \left\{I(Z_{i}(t_{0})=k)-p_{k}(t_{0}|X_{i})\right\}\;. \end{array}$$

Each term has a weight reflecting the probability $W_{i}{\left(t\right)}^{-1}=\mathrm{Pr}(\overset{*}{\underset{i}{C}}>t|X_{i})$ of being uncensored, and typically $A(\beta ,X_{i})$ contains the partial derivatives of $p_{k}(t_{0}|X_{i})=g^{-1}\left(\beta^{\text{T}}X_{i}\right)$ with respect to β. Clearly, this approach requires a model for the random censoring $C^{*}$, and in its simplest form, the resulting weights could be given by the Kaplan‐Meier estimator. However, if covariates affect censoring, a regression model would be needed for estimating the weights. The terms in (15) are independent, and a sandwich estimator for the variance of the solution to (15), denoted by ${\hat{\beta }}_{D}$, is typically used, with a contribution arising from the need to estimate $W_{i}(\cdot )$ [45].

The marginal regression model (14) can also be analyzed using PV. With this approach, an outcome variable for each observation i, to be used in a GEE, is computed via a *base estimator* of the marginal state occupancy probability $p_{k}(t_{0})$ (ignoring covariates), denoted by ${\hat{p}}_{k}(t_{0})$. The Aalen‐Johansen estimator is consistent, even without assuming the multistate process is Markov [29, 51]. The PV for subject i is given by

(16) $$\begin{array}{ll} V_{i} & =n{\hat{p}}_{k}(t_{0})-(n-1){\hat{p}}_{k}^{\left(-i\right)}(t_{0})\\ & ={\hat{p}}_{k}(t_{0})+(n-1)\left\{{\hat{p}}_{k}(t_{0})-{\hat{p}}_{k}^{\left(-i\right)}(t_{0})\right\}\;, \end{array}$$

where ${\hat{p}}_{k}^{\left(-i\right)}$ is the (Aalen‐Johansen) estimator applied to the sample of size n−1 obtained by removing subject i from the full sample. The intuition is that $V_{i}$ quantifies the extent to which the base estimator ${\hat{p}}_{k}(t_{0})$ is affected by data from subject i, and in the special case of no censoring (where the Aalen‐Johansen estimator reduces to the relative frequency $\sum_{i=1}^{n}I(Z_{i}(t_{0})=k)/n$ of processes in state k at time $t_{0}$), $V_{i}$ is simply $I(Z_{i}(t_{0})=k)$ [37]. Note that $V_{i}$ is calculated by (16) for *all* the subjects, even if $I(Z_{i}(t_{0})=k)$ is observed. The PV is then used as response in a GEE, $U_{P}(\beta )=0$, where

(17) $$U_{P}(\beta )=\overset{n}{\underset{i=1}{\sum }}A(\beta ,X_{i})\left\{V_{i}-p_{k}(t_{0}|X_{i})\right\}.$$

The terms in $U_{P}(\beta )$ are not independent [52], and special techniques are needed for evaluating large‐sample properties as n→∞ of ${\hat{\beta }}_{P}$, the solution to $U_{P}(\beta )=0$. These depend on the properties of the influence function of the functional (denoted ϕ) that maps the data from the observed multistate process onto ${\hat{p}}_{k}(t_{0})$ [52, 53]. A necessary condition for the properties to hold is that censoring does not depend on covariates. If covariates affect censoring, the Aalen‐Johansen base estimator in (16) may be replaced by an IPCW estimator of $p_{k}(t_{0})$ [54]. It should be noted that the required properties of the influence function are typically not fulfilled when the base estimator is based on data with delayed entry [55]. Another consequence of the lack of independence among the terms in (17) is that the standard GEE sandwich estimator for the variance of ${\hat{\beta }}_{P}$ should be replaced by a corrected estimator that also involves the second‐order influence function of the functional ϕ. However, in practical applications, the correction terms tend to be small [52].

No systematic comparison between the estimators ${\hat{\beta }}_{D}$ and ${\hat{\beta }}_{P}$, based on (15) or (17), has been conducted, although such a comparison has been studied in the special case where the base estimator is Kaplan‐Meier [56]. In large samples, the computation of PV using (16) can be time‐consuming. Approximations via *infinitesimal jackknife* PV method [55], as implemented in the survival package in R, offer a more efficient alternative. Additionally, for certain specific multistate models, such as those shown in Figure 1a,b, and f, direct models for all time points are available. These models each require specialized estimating equations, such as those based on partial likelihood principles [57, 58, 59, 60].

## 4 Intermittent Observation of Continuous‐Time Processes

In many settings, transitions between states are not directly observed, and only the state occupied at intermittent assessment times is recorded. Examples include studies of retinopathy where visual acuity is assessed during clinic visits [61], diabetic hepatology [62] where liver function is evaluated through blood tests or biopsies, and studies of osteoporosis where periodic radiographic examinations can detect asymptomatic vertebral fractures [63]. To accommodate intermittent observation in the likelihood construction, we consider the multistate process $\left\{Z_{i}(s),0\leq s\right\}$ along with time‐independent covariates $X_{i}$. The assessment process is represented by a counting process $\left\{A_{i}(s),0\leq s\right\}$, which records the number of assessments up to time s. An assessment at time t results in $dA_{i}(t)=A_{i}(t)-A_{i}(t^{-})=1$, while $dA_{i}(t)=0$ otherwise. Since visits can only occur for individuals still on the study, the assessment process is terminated at $C_{i}$ so we observe $d{\bar{A}}_{i}(t)=Y_{i}(t)dA_{i}(t)$ and ${\bar{A}}_{i}(t)=\int_{0}^{t}d{\bar{A}}_{i}(s)$. If ${\bar{ℋ}}_{i}(t)=\{Y_{i}(s),{\bar{A}}_{i}(s),d{\bar{N}}_{i}(s),0\leq s<t,X_{i}\}$, then the assessment‐process intensity is

$$\underset{\Delta t↓0}{\lim}\;\frac{1}{\Delta t}\mathrm{Pr}\left(\Delta A_{i}(t)=1|{\bar{ℋ}}_{i}(t)\right)$$

where $\Delta A_{i}(t)=A_{i}({\left(t+\Delta t\right)}^{-})-A_{i}(t^{-})$. This general formulation involves a dependence on $\left\{Z_{i}(s),0\leq s<t\right\}$, but this process is not fully observed. In such cases, joint models for the disease and visit processes must be specified. These models are often constructed under the assumption of conditional independence given latent variables with an assumed distribution. Lange et al. [64] and Cook and Lawless [65] propose joint models that account for local dependence in which the visit intensity may depend on the state occupied at $t^{-}$ and discuss the independence conditions needed to focus on the partial likelihood contributions involving only the intensities of the multistate process; see also Grüger et al. [66]. Assume individual i has $m_{i}$ visits at times $0\leq a_{i0}<a_{i1}<\cdots <a_{im_{i}}$ and let ${\bar{ℋ}}_{i}^{\circ }(t)=\{Y_{i}(s),{\bar{A}}_{i}(s),0\leq s<t,(Z_{i}(a_{r}),a_{r}),r=0,1,\ldots ,{\bar{A}}_{i}(t^{-}),X_{i}\}$represent the observed history of individual i at time t. If the visit process is noninformative, meaning no parameters are shared between the visit and multistate models, we can ignore the visit process and focus on a likelihood of the form

(18) $$\overset{m_{i}}{\underset{r=1}{\prod }}\mathrm{Pr}\left(Z_{i}(a_{r})|a_{r},{\bar{ℋ}}_{i}^{\circ }(a_{r})\right).$$

As discussed earlier, expressing $\mathrm{Pr}\left(Z_{i}(a_{r})|a_{r},{\bar{ℋ}}_{i}^{\circ }(a_{r})\right)$ in terms of intensity functions can be challenging for general processes, but Markov models are relatively easy to handle. For instance, if $\lambda_{kl}(t|X_{i})=\lambda_{kl}(X_{i})=\lambda_{kl}\exp(X_{i}^{\text{T}}\beta_{kl})$ for all k→l transitions, we can construct the transition intensity matrix $Q(X_{i})$, where the off‐diagonal entries are given by $\lambda_{kl}(X_{i})$ and the diagonal entries are $-\sum_{l=0,l\neq k}^{K}\lambda_{kl}(X_{i})$. Then, the transition probability matrix $P(s,t|X_{i})=\exp\{Q(X_{i})(t-s)\}$ has entries $p_{kl}(s,t|X_{i})=\mathrm{Pr}(Z_{i}(t)=l|Z_{i}(s)=k,X_{i})$. Kalbfleisch and Lawless [67] discuss a Fisher‐scoring algorithm for such models, which, along with other optimization methods, is implemented in the msm [23] package in R. The assumption of time‐homogeneous baseline intensities can be relaxed to allow them to be piecewise‐constant rates upon specifying the number and location of cut points.

Titman [68, 69] considers semi‐Markov models with sojourn times having phase‐type distributions [70]; see also Yang et al. [71] Satten [72] considers progressive models with Markov intensities given a common multiplicative random effect which accommodates both serial dependence in the sojourn times and heterogeneity across individuals in the progression rate. Extensions to accommodate more general forms of heterogeneity have also been developed to include higher dimensional random effects [73] and mover‐stayer components [74]. Random effect (or frailty) models are particularly useful for processes observed intermittently, where history dependence is suspected but detailed information is lacking, making direct dependence modeling challenging.

Transition information is often available through dual observation schemes. For instance, in dementia studies that model cognition and survival, cognitive state is observed only during assessments, while survival times are recorded continuously. A simple model to illustrate this setup is the illness‐death model shown in Figure 1c. If the assessment process for state 1 involves random visit times, the entry time to state 1 is interval‐censored. Additionally, in some studies, there may be uncertainty about whether a transition has occurred. For example, if the disease had not been diagnosed by the last visit, it is unclear whether it developed between that visit and the time of death. Therefore, the likelihood must be adjusted to account for this uncertainty. In this context, Leffondre et al. [75] explore the usefulness of the illness‐death model when the primary interest is overall survival. Joly et al. [76] develop methods for fitting intensity‐based models to such data, using spline‐based approaches for modeling the intensities or the Weibull form [77].

The inclusion of time‐dependent explanatory variables requires additional assumptions when these variables are not measured continuously. As a result, this situation is typically addressed using time‐dependent but interval‐constant covariates, where the times of changes in value are known. Boruvka and Cook [78] examine identifiability issues and apply a sieve maximum likelihood approach to estimate transition intensities and covariates' effects. More generally, Commenges et al. [79] explore the estimation of multistate processes under intermittent observation using splines. For further discussion of time‐dependent covariates in multistate models, see [16, 31].

### 4.1 The Psoriatic Arthritis Data Revisited

**FIGURE 4:**
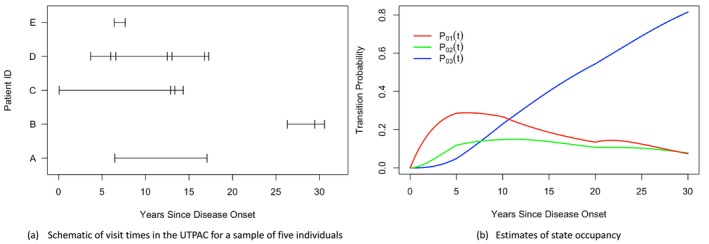
Joint damage data from the University of Toronto Psoriatic Arthritis Cohort: sample data and estimates of state occupancy based on fitted Markov models with piecewise‐constant baseline intensities having cut‐points at 5, 10, and 20 years from the onset of psoriatic arthritis. (a) Schematic of visit times in the UTPAC for a sample of five individuals. (b) Estimates of state occupancy.

Here, we analyze the joint damage data in psoriatic arthritis from Section 1.2.2. Patients in this registry are scheduled for annual clinical examinations and biennial radiographic exams, but visit times vary greatly across and within patients. Figure 4a shows raw data from five patients, with horizontal lines representing the period from the first clinic visit to loss to follow‐up or death. Vertical hatch marks indicate radiographic exams, highlighting the significant variability in the frequency of imaging data collection among individuals. We consider a four‐state model from Gladman and Farewell [22], as shown in Figure 1b. Specifically, state 0 corresponds to having no damaged joints, state 1 to 1–4 damaged joints, state 2 to 5–9 damaged joints, and state 3 is an absorbing state representing 10 or more damaged joints. The time scale starts at the age of psoriatic arthritis diagnosis. Assuming a noninformative visit process, we use the likelihood (18) to estimate piecewise‐constant transition intensities under Markov models, with cut‐points at 5, 10, and 20 years after disease onset. Figure 4b and Table 2 are based on the code provided in Section S3 of the Supporting Information file.

**TABLE 2:** Estimates from fitting multiplicative intensity‐based Markov models to data on joint damage from the University of Toronto Psoriatic Arthritis Cohort with piecewise‐constant baseline intensities having cutpoints at 5, 10, and 20 years from the onset of psoriatic arthritis.

**(a)** Estimated regression coefficients (Est) and relative risk (RR)						
Transition	Covariate	Est	SE	RR	95% CI	p
0→1	Effusions	0.742	0.400	2.100	(0.960, 4.597)	0.063
	Elevated ESR	0.239	0.278	1.271	(0.737, 2.188)	0.389
1→2	Effusions	0.536	0.297	1.710	(0.955, 3.062)	0.071
	Elevated ESR	0.774	0.281	2.169	(1.250, 3.759)	0.006
2→3	Effusions	0.306	0.311	1.358	(0.739, 2.497)	0.325
	Elevated ESR	−0.359	0.364	0.698	(0.342, 1.425)	0.323

**(b)** Estimated transition intensities (Int), baselines are with covariates set to 0								
	Baseline		[5,10)		[10,20)		[20,∞)	
Transition	Int	95% CI	Int	95% CI	Int	95% CI	Int	95% CI
0→1	0.092	(0.052, 0.161)	0.718	(0.387, 1.333)	0.419	(0.187, 0.939)	1.566	(0.763, 3.215)
1→2	0.097	(0.048, 0.198)	0.828	(0.394, 1.743)	0.796	(0.406, 1.559)	1.126	(0.502, 2.529)
2→3	0.244	(0.072, 0.821)	1.326	(0.434, 4.048)	1.174	(0.381, 3.618)	1.373	(0.431, 4.375)

Figure 4b is obtained by maximizing the likelihood with respect to all intensity parameters $\lambda ={\left(\lambda_{01}^{\text{T}},\lambda_{12}^{\text{T}},\lambda_{23}^{\text{T}}\right)}^{\text{T}}$ (while omitting covariates) using the msm() package, where each $\lambda_{kl}$ is a 4×1 vector of parameters of the piecewise‐constant intensity for a k→l transition with cut‐points at 5, 10, and 20 years since disease diagnosis. Using these estimates, we compute $P(0,t;\hat{\lambda })$ and plot $p_{k}(t;\hat{\lambda })$ over the 30 years following disease onset. The state occupancy probabilities for the transient states rise and then fall as patients progress to more severe joint damage, with nearly 50% expected to reach 10 or more damaged joints after 20 years. Table 2 summarizes the results of intensity‐based regression models, including baseline covariates‐an indicator of extensive effusions and an indicator of an elevated erythrocyte sedimentation rate (ESR), a marker of inflammation. Table 2a shows the regression coefficient for each transition, and Table 2b provides the estimated transition intensities for each time interval. Interestingly, an elevated ESR at baseline is a strong predictor of a faster transition from 1→2, but it does not significantly impact the other two transitions.

## 5 Multistate Models With Frailty or Copula Approaches

At times, even after accounting for covariates explaining between‐individual variation in disease course, life trajectories display greater variability than anticipated based on model assumptions. To address this, models with latent random effects can be considered—in survival analysis these random effects are called frailties [80]. Frailties are often introduced to model dependence but dependence can also be modeled using copula functions which are multivariate distribution functions with uniform marginal distributions [81]. More general joint distributions are formulated from copula functions through probability integral transformations of respective failure time variables [82]. Much attention has been given to use of frailty‐based and copula‐based models for dependence within individuals or clusters. Within the framework of multistate survival models, two key scenarios are relevant:

i. *Within‐subject dependence*: Here, random effects or copula models account for unobserved covariates that influence event times within the same individual. For instance, in Figure 1d, a random effect might explain the unobserved dependence between the transition times of 0→1 and $1\to 2^{\prime }$.
ii. *Between‐subjects dependence*: In this case, clustered data, such as families or study centers, involve correlated failure times among individuals within the same cluster. Random effects or copula models can address this unobserved dependence.

Both scenarios are discussed in what follows and we focus on frailty models. Applying frailty or copula models in the general multistate framework can be complex, as unobserved heterogeneity may vary across different transitions. We therefore focus primarily on the illness‐death model (Figure 1c or d) for simplicity. As discussed in Sections 5.1 and 5.2, within‐ and between‐subject dependence is an important area warranting further methodological research.

### 5.1 Within‐Subject Random Effect

Consider, for example, the illness‐death model for a chronic disease, which involves three possible transitions: healthy to disease (0→1), healthy to disease‐free death (0→2), and disease to post‐disease death ($1\to 2^{\prime }$). Survival after diagnosis is left‐truncated by the diagnosis time, and it is often unrealistic to assume that observed covariates capture all dependence between diagnosis time and post‐diagnosis death time. This motivates including an unobserved subject‐specific random effect to account for residual dependence among a subject's event times.

The following discussion examines two distinct approaches: one focuses on the regression coefficients of the observed covariates conditioned on the unobserved random effects, while the other considers the regression coefficients of the observed covariates without conditioning on the unobserved random effects. Each approach provides a different interpretation of the effect of the observed covariates, as detailed below. These approaches will be demonstrated using the Cox model (Section 5.1.1) and the accelerated failure time model (Section 5.1.2). Software implementation is outlined in Section 6.7.

Random‐effects models for failure time outcomes are commonly referred to as frailty models, where a random subject‐specific effect—representing unobserved risk factors associated with failure times—is termed the frailty. Xu et al. [83] proposed a one‐parameter gamma‐frailty model, in which a single frailty variate is shared across all three transitions of the illness‐death model (see Equations ((19), (20), (21)) below for details). This modeling strategy has been widely adopted in subsequent work [39, 84, 85, 86, 87, 88], and offers a parsimonious framework; at the same time, the assumption of a common unobserved risk structure across all transitions may not be suitable for every application. In more complex multistate models involving additional transitions, the assumption of a single frailty variate may provide an oversimplified representation of the underlying heterogeneity. Alternatives include the use of subject‐specific vector‐valued frailty terms [16, 89], or transition‐specific transformations of a shared frailty component [89]. Most existing frailty‐based approaches for multistate models have focused on illness‐death and progressive (forward) processes, such as in Figure 1b, and include extensions accommodating competing risks and hierarchical clustering structures [90].

A key feature of the frailty approach is the assumption that, given the observed covariates and the frailty variate, individual transition times are independent (or quasi‐independent [39]). This assumption allows for the separate modeling of each transition. Also, it is often assumed that the unobserved frailty variate and the observed covariates are independent. However, certain assumptions are required to ensure model identifiability [91], and verifying these assumptions with available data can be challenging. When addressing unobservable random effects, it is important to distinguish between two approaches: conditional modeling, which conditions on both observed covariates and the unobserved frailty variate, and marginal (population‐average) modeling, which conditions only on the observed covariates. In linear models, if the observed covariates are independent of the frailty variate, these approaches yield the same estimates. However, in nonlinear models, they do not, making the distinction practically significant. The choice between the two approaches depends on the specific objectives of the analysis.

The choice of time scale in multistate models with frailty is crucial. Consider two illness‐death models: in the first, the states are “Healthy,” (state 0) “Disease,” (1) and “Death” (2 and $2^{\prime }$). In the second, the states are “Surgery,” “Recurrence,” and “Death.” In the first case, a negative association is expected between age at diagnosis and the time from diagnosis to death, as older individuals generally have shorter lifespans post‐diagnosis. Therefore, given our intention to utilize a shared random effect to capture the interplay between the three transitions, it is natural to utilize the age scale for all three transitions. In the second case, a clock‐reset approach is needed (i.e., semi‐Markov), where time resets at each transition, in case early recurrence is positively associated with the remaining lifespan. Here, the time scale for each transition depends on the time spent in the preceding state.

#### 5.1.1 Within‐Subject Random Effects: Conditional versus Marginalized Illness‐Death Cox Models

Since the illness‐death model of Figure 1d describes a progressive process with each state visited at most once, we adopt the simplified notation of Section 1, and for n independent observations, let $T_{i1}$ and $T_{i2}$ denote the times to non‐terminal (e.g., disease diagnosis) and terminal (e.g., death) events, respectively, i=1,…,n. The joint distribution of $\left(T_{i1},T_{i2}\right)$ is supported over $t_{2}\geq t_{1}$. For individuals who experience the terminal event before the non‐terminal event, we set $T_{i1}=\infty$. Let $C_{i}$ represent the right‐censoring time, and $X_{i}$ a set of time‐fixed covariates. While some of the models discussed here can be easily extended to incorporate time‐dependent covariates, others would require substantial additional modifications.

Define $W_{i1}=\min(T_{i1},T_{i2},C_{i})$. Let $\delta_{i1}=I(W_{i1}=T_{i1})$ indicate whether $W_{i1}$ corresponds to the non‐terminal event, and $\delta_{i2}=I(W_{i1}=T_{i2})$ indicate whether it corresponds to the terminal event. Also, let $W_{i2}=\delta_{i1}\min(T_{i2},C_{i})$ be the time of the terminal event or censoring, when the non‐terminal event is observed, and 0 otherwise. Let $\delta_{i3}=\delta_{i1}I(W_{i2}=T_{i2})$ indicate whether the terminal event was observed after the non‐terminal event. The observed data are $\left\{W_{i1},W_{i2},\delta_{i1},\delta_{i2},\delta_{i3},X_{i},;,i=1,\ldots ,n\right\}$. We consider a scalar latent frailty variable $\omega_{i}>0$ for individual i, with cumulative distribution $F_{\omega ;\theta }$ indexed by an unknown parameter θ.

Xu et al. [83] proposed an illness‐death model featuring three Cox‐based hazard functions. One of their key innovations was the inclusion of a gamma‐distributed shared frailty variate, which acts multiplicatively on each intensity function. This accommodates unobserved dependencies between the non‐terminal and terminal event times. With time‐independent covariates and ${ℋ}_{i}(t)=\{Z_{i}(s),0<s<t,X_{i}\}$, the conditional intensity functions (given $\omega_{i}$) governing the three transitions are expressed as

(19) $$\begin{array}{ll} & \lambda_{01}\left(t|Z_{i}(t^{-})=0,{ℋ}_{i}(t),\omega_{i}\right)\\ & \;=\underset{\Delta t\to 0}{\lim}{\left(\Delta t\right)}^{-1}\mathrm{Pr}\left(\Delta N_{i01}(t)=1|Z_{i}(t^{-})=0,X_{i},w_{i}\right)\\ & \;=\underset{\Delta t\to 0}{\lim}{\left(\Delta t\right)}^{-1}\mathrm{Pr}\left(t\leq T_{i1}<t+\Delta t|T_{i1}\geq t,T_{i2}\geq t,X_{i},\omega_{i}\right)\\ & \;=\omega_{i}\lambda_{01}(t)\exp\left(X_{i}^{\text{T}}\beta_{01}\right)\;\;,\;\;t>0 \end{array}$$

(20) $$\begin{array}{ll} & \lambda_{02}\left(t|Z_{i}(t^{-})=0,{ℋ}_{i}(t),\omega_{i}\right)\\ & \;=\underset{\Delta t\to 0}{\lim}{\left(\Delta t\right)}^{-1}\mathrm{Pr}\left(\Delta N_{i02}(t)=1|Z_{i}(t^{-})=0,X_{i},w_{i}\right)\\ & \;=\underset{\Delta t\to 0}{\lim}{\left(\Delta t\right)}^{-1}\mathrm{Pr}\left(t\leq T_{i2}<t+\Delta t|T_{i1}\geq t,T_{i2}\geq t,X_{i},\omega_{i}\right)\\ & \;=\omega_{i}\lambda_{02}(t)\exp\left(X_{i}^{\text{T}}\beta_{02}\right)\;\;,\;\;t>0 \end{array}$$

and for s>t>0

(21) $$\begin{array}{ll} & \lambda_{12^{\prime }}\left(s|Z_{i}(s^{-})=1,{ℋ}_{i}(s),\omega_{i}\right)\\ & \;=\underset{\Delta s\to 0}{\lim}{\left(\Delta s\right)}^{-1}\mathrm{Pr}(\Delta N_{i12}(s)=1|T_{i1}=t,Z_{i}(s^{-})=1,X_{i},w_{i})\\ & \;=\underset{\Delta s\to 0}{\lim}{\left(\Delta s\right)}^{-1}\mathrm{Pr}\left(s\leq T_{i2}<s+\Delta s|T_{i1}=t,T_{i2}\geq s,X_{i},\omega_{i}\right)\\ & \;=\omega_{i}\lambda_{12^{\prime }}(s)\exp\left(X_{i}^{\text{T}}\beta_{12^{\prime }}\right) \end{array}$$

where $\lambda_{kl}(\cdot )$ and $\beta_{kl}$, $kl\in \{01,02,12^{\prime }\}$, are transition‐specific baseline hazard functions and regression coefficients, respectively. Given that subject i was diagnosed (i.e., made 0→1 transition) at time $T_{i1}=t$, $T_{i2}>t$, the post diagnosis death time $T_{i2}$ is left truncated by t. The time to the non‐terminal event, $T_{i1}=t$, is not incorporated into the covariate vector for $\lambda_{12^{\prime }}(s|{ℋ}_{i}(s),\omega_{i})$. Instead, the dependence between the potential event times $T_{i1}$ and $T_{i2}$ is derived from two key factors: the so‐called explanatory hazard ratio $\lambda_{12^{\prime }}(t_{2})\exp(X_{i}^{\text{T}}\beta_{12^{\prime }})/\{\lambda_{02}(t_{2})\exp(X_{i}^{\text{T}}\beta_{02})\}$ [83] and the latent frailty distribution parameter θ. The explanatory hazard ratio characterizes the local dependence between the non‐terminal and terminal events times not captured by the frailty. Should $T_{i1}$ and $T_{i2}$ be independent, the explanatory hazard ratio is constant at 1, and the frailty variate is also constant at 1 (i.e., the frailty distribution is degenerated).

Estimation of models ((19), (20), (21)) under gamma‐distributed frailty with mean 1 and unknown variance can be performed by semiparametric maximum‐likelihood estimators (MLE), where the likelihood is obtained by averaging the *conditional* likelihood of observed data, given $\omega_{1},\ldots ,\omega_{n}$, over the distribution of $\omega_{i}$ [83]. A semiparametric Bayesian approach [84] can alternatively be used. Interesting modifications of ((19), (20), (21)) are found in Jiang and Haneuse [87] and Lee et al. [92].

Survival predictions based on the conditional hazards (i.e., given $\omega_{i}$) given by ((19), (20), (21)) require knowledge of the unobserved frailty variate, which limits their practical applicability. By integrating out the frailty, we obtain the so‐called *marginalized hazards* with respect to $\omega_{i}$. These marginalized hazards depend strongly on the assumed frailty distribution and its parameters. Although this approach allows prediction without directly observing $\omega_{i}$, it introduces sensitivity to frailty misspecification and complicates the interpretation of covariate effects. To address these issues, Gorfine et al. [39] proposed an alternative strategy for frailty‐based illness‐death models, where the marginal hazards with respect to $\omega_{i}$ are modeled using Cox models, and the conditional hazards (given $\omega_{i}$) necessary to yield this proportional specification are derived for a specified frailty distribution. These conditional hazards, which depend on $\left(X_{i},\omega_{i}\right)$ and the parameters of the marginal hazards, depart from the proportional‐hazards structure. This approach allows for the estimation of marginal model parameters while incorporating frailties to account for unobserved subject‐specific covariates. Specifically, the conditional hazards of the illness‐death model given $\omega_{i}$ are given by

(22) $$\lambda_{0l}(t|Z_{i}(t^{-})=0,{ℋ}_{i}(t),\omega_{i})=\omega_{i}\alpha_{0l}(t|X_{i})\;\;,\;\;l=1,2\;\;,\;\;\;t>0$$

(23) $$\lambda_{12^{\prime }}(s|Z_{i}(s^{-})=1,{ℋ}_{i}(s),\omega_{i})=\omega_{i}\alpha_{12^{\prime }}(s|X_{i})\;\;,\;\;s>t>0.$$

The corresponding marginalized hazard functions with respect to $\omega_{i}$ are defined

(24) $$\lambda_{0l}(t|Z_{i}(t^{-})=0,{ℋ}_{i}(t))=\lambda_{0l}(t)\exp\left(X_{i}^{\text{T}}\beta_{0l}\right)\;\;,\;\;l=1,2\;\;,\;\;t>0$$

(25) $$\lambda_{12^{\prime }}(s|Z_{i}(s^{-})=1,{ℋ}_{i}(s))=\lambda_{12^{\prime }}(s)\exp\left(X_{i}^{\text{T}}\beta_{12^{\prime }}\right)\;\;,\;\;s>t>0$$

with unspecified baseline hazard functions $\lambda_{kl}(\cdot )$, $kl\in \{01,02,12^{\prime }\}$. To maintain the relationship between marginalized and conditional hazard functions, the conditional hazards must be mapped to their marginalized counterparts according to the assumed frailty distribution. Indeed, the nonnegative functions $\alpha_{kl}(\cdot |X_{i})$ are determined by the distribution of the frailty $\omega_{i}$ and the corresponding marginalized hazard $\lambda_{kl}(\cdot |Z_{i}(\cdot ),{ℋ}_{i}(\cdot ))$. For example, under the gamma‐frailty model with expectation 1 and variance θ, it can be shown that $\alpha_{kl}(t|X_{i})=\lambda_{kl}(t)\alpha_{kl}^{*}(t|X_{i})$, kl∈{01,02,12}, where $\alpha_{0l}^{*}(t|X_{i})=\exp\left(X_{i}^{T}\beta_{0l}\right)\exp\left\{\theta \Lambda_{0.}(t|X_{i})\right\}$, l=1,2, $\Lambda_{0.}(t|X_{i})=\Lambda_{01}(t|X_{i})+\Lambda_{02}(t|X_{i})$ and $\alpha_{12^{\prime }}^{*}(t|X_{i})=\exp\left(X_{i}^{\text{T}}\beta_{12^{\prime }}\right)\exp\left\{\Lambda_{12^{\prime }}(t|X_{i})\theta /(1+\theta )\right\}/(1+\theta )$. In summary, the above frailty‐based models (22) and (23) that depart from a proportional hazards structure are expressed in terms of the main parameters of interest, $\beta_{kl}$ and $\lambda_{kl}(\cdot )$ of models (24) and (25). The frailty‐based models are particularly appealing for developing estimation procedures, as they assume that, given the observed covariates and the frailty variate, $T_{i1}$ and $T_{i2}$ are quasi‐independent. A pseudo‐likelihood approach was developed for estimating the parameters and their standard errors, under standard frailty‐specific assumptions [39].

#### 5.1.2 Within‐Subject Random Effect: Additive versus Multiplicative Illness‐Death AFT Models

An important alternative to intensity‐based modeling of multistate processes is the adaptation of accelerated failure time (AFT) regression techniques. Unlike multiplicative intensity‐based models, AFT models parameterize covariate effects directly on the time scale, offering a distinct and often more interpretable characterization of covariate effects [93]. In competing risks, recurrent events and multivariate‐failure time settings, AFT models are typically formulated in terms of latent failure times corresponding to different transitions, acknowledging that some of these latent event times may not be observed due to the occurrence of competing events; see, for example [94, 95, 96, 97]. This latent‐time formulation is well established. In this section, we review recent developments of AFT models for illness–death processes, with particular emphasis on extensions incorporating random effects to account for unobserved heterogeneity.

Lee et al. [86] proposed the following *additive scale‐change* model

$$\begin{array}{ll} \log(T_{i1}) & =X_{i}^{\text{T}}\beta_{01}+\omega_{i}+U_{i01}\;,\;T_{i1}>0\\ \log(T_{i2}) & =X_{i}^{\text{T}}\beta_{02}+\omega_{i}+U_{i02}\;,\;T_{i2}>0\;,\\ & \;\text{if subject}\;i\;\text{does not experience the non‐terminal event}\\ \log(T_{i2}) & =X_{i}^{\text{T}}\beta_{12^{\prime }}+\omega_{i}+U_{i12^{\prime }}\;,\;T_{i2}>T_{i1}>0,\\ & \;\;\text{if subject}\;i\;\text{experiences the non‐terminal event.} \end{array}$$

where $X_{i}$ has 1 in its first component to allow for an intercept. The term “additive” refers to the inclusion of a shared‐frailty term, $\omega_{i}$, that enters additively in the log linear specification across all transitions. The model is defined in terms of latent, path‐specific failure times, with the observed terminal time determined by the realized transition path. The model aligns with typical illness‐death progression: a subject first experiences an event and its type determines the subsequent transition. If the failure corresponds to the non‐terminal event, the subsequent transition time $T_{i2}$ is modeled according to the path from state 1 to state $2^{\prime }$. The random errors $U_{ikl}$ are independent across i=1,…,n and are transition specific for $kl\in \{01,02,12^{\prime }\}$, possibly having unspecified distributions. The association between $T_{i1}$ and $T_{i2}$ is determined by the distribution of $\omega_{i}$, manifesting as an additive component in the log‐failure times scale. Given the assumption of $\omega_{i}$ following a normal distribution, both parametric and semiparametric Bayesian estimation methods are available [86].

Alternatively, a *multiplicative* frailty‐based AFT model has been proposed [88], in which the unobserved frailty variate is not directly expressed in the log‐failure time linear model. Instead, it influences the distribution of the random errors $U_{ikl}$. In particular, the model is defined by

$$\begin{array}{ll} \log(T_{i1}) & =X_{i}^{\text{T}}\beta_{01}+U_{i01}\;,\;T_{i1}>0\\ \log(T_{i2}) & =X_{i}^{\text{T}}\beta_{02}+U_{i02}\;,\;T_{i2}>0\;,\;\\ & \;\text{if subject}\;i\;\text{does not experience the non‐terminal event}\\ \log(T_{i2}) & =X_{i}^{\text{T}}\beta_{12^{\prime }}+U_{i12^{\prime }}\;,\;T_{i2}>T_{i1}>0\;,\;\\ & \;\text{if subject}\;i\;\text{experiences the non‐terminal event.} \end{array}$$

The dependence between $T_{i1}$ and $T_{i2}$ is incorporated via the following shared frailty model. Given individual i's frailty variate $\omega_{i}$, it is assumed that the respective conditional baseline hazard functions of $\exp(U_{ikl})$, $kl\in \{01,02,12^{\prime }\}$, are given by

$$\begin{array}{ll} & \lambda_{01}(t|\omega_{i})=\omega_{i}\lambda_{01}(t)\;,\;t>0\\ & \lambda_{02}(t|\omega_{i})=\omega_{i}\lambda_{02}(t)\;,\;t>0\;,\\ & \;\text{if subject}\;i\;\text{does not experience the non‐terminal event}\\ & \lambda_{12^{\prime }}(s|t_{1},\omega_{i})=\omega_{i}\lambda_{12^{\prime }}(s)\;,\;s>t_{1}>0\;,\\ & \;\text{if subject}\;i\;\text{experiences the non‐terminal event.} \end{array}$$

where each $\lambda_{kl}(\cdot )$ is an unspecified baseline hazard function of $\exp(U_{il})$. Importantly, this model offers a clear conceptual separation between observed covariate effects on event times and unobserved heterogeneity captured through the frailty. Under the assumption that $\omega_{i}$ are gamma distributed with mean 1 and unknown variance θ, a semiparametric MLE (based on a kernel‐smoothed likelihood combined with an EM algorithm) is available [88]. Section 2.2 of Kats and Gorfine [88] delves into the conceptual distinctions between the above additive and multiplicative approaches. It is elucidated that the hazards of the additive approach may exhibit non‐monotonic behavior with respect to $\omega_{i}$. In contrast, the hazards of the multiplicative model demonstrate monotonic increase as a function of $\omega_{i}$ across all error distributions. Consequently, the multiplicative‐frailty model offers a simpler interpretation.

#### 5.1.3 The Rotterdam Tumor Bank Data Revisited

The Rotterdam tumor bank data (Section 1.2.3) were analyzed in Kats and Gorfine [88] using three frailty‐based models: the multiplicative AFT model [88], the marginalized Cox model [39], and the conditional Cox model [84]. The additive‐frailty AFT model [86], implemented in the R package SemicompRisks, models the time $T_{i2}-T_{i1}$ for subjects who experience death following relapse. However, this model encountered convergence issues when applied to the Rotterdam data. (see [88] for further details). In the current analysis, we extend this evaluation by comparing eight models: four based on the Cox framework and four based on the AFT framework. The Cox‐based models include the marginalized Cox model [39] and the following three models without frailty:

1. Cox without frailty I: Three separate Cox models are fitted for each of the transitions. For the competing risks transitions 0→1 and 0→2 (relapse and death), estimation proceeds by treating the competing event as right‐censored. For the transition $1\to 2^{\prime }$, left truncation by relapse time is handled naively using standard risk‐set adjustment.
2. Cox without frailty II: This model extends Model I by adding the standardized relapse time as a time‐independent covariate in the $1\to 2^{\prime }$ transition model. Specifically, let $V_{i}=T_{i1}$ denote the relapse time (entry into state 1) for subject i, and define $SV_{i}=(V_{i}-\bar{V})/S_{V}$, where $\bar{V}$ and $S_{V}$ are the sample mean and standard deviation of $\left\{V_{i}:\Delta_{i1}=1\right\}$, that is, computed among individuals with observed relapse. This centering and scaling places relapse time on a comparable numerical scale and facilitates interpretation of regression effects. The inclusion of $SV_{i}$ follows [98] and is intended to address the dependent left truncation induced by relapse.
3. Cox without frailty III: An extension of Model II, this model includes a linear truncated spline with four knots (at the 20th, 40th, 60th, and 80th percentiles) to flexibly model nonlinear effects of the standardized relapse time. Specifically, it incorporates terms of the form $\sum_{j=1}^{4}\gamma_{j}(SV_{i}-k_{j})I(SV_{i}\geq k_{j})$, where $\gamma_{j}$, j=1,…,4, are regression coefficients.

A parallel set of analyses was performed using four AFT‐based models. The results are summarized in Tables 3 and 4. A comparison of these tables highlights how covariate effects differ across Cox‐based and AFT‐based modeling frameworks, particularly in their interpretation, magnitude, and statistical significance. In Cox models, coefficients are log‐hazard ratios (HRs), with HR > 1 indicating increased hazard and HR < 1 indicating protective effect. In AFT models, coefficients are log‐acceleration factors, where values > 0 imply prolonged time to event (i.e., reduced hazard), and values < 0 indicate shorter times to event (i.e., increased hazard). Specifically,

- Treatment Effect (Hormonal and/or Chemotherapy): In Cox models, treatment is significantly associated with reduced hazard of transition from state 0 to 1, confirming a protective effect on recurrence. In AFT models, the same treatment is associated with positive coefficients, indicating a significant delay in time to recurrence—consistent with the Cox results but on a different time scale. This effect is particularly strong in the multiplicative frailty model, suggesting that adjusting for unobserved heterogeneity improves inference precision.
- Tumor Size and Grade: In the Cox framework, larger tumor size and higher grade show significantly increased hazard for recurrence and mortality post‐recurrence, reflecting worse prognosis. In the AFT models, these same covariates generally have negative coefficients, confirming their association with shorter survival or quicker relapse. However, effect estimates differ across frameworks (HR versus acceleration), and in some AFT fits they appear closer to the null, especially without frailty.
- Number of Positive Lymph Nodes: This variable is strongly predictive in both tables. In the Cox models, it shows a consistent and significant increase in hazard across transitions. In the AFT models, it corresponds to significantly negative coefficients, indicating shorter times to recurrence and death, again confirming its prognostic importance.
- Estrogen and Progesterone Receptors: In Table 3, higher receptor levels are associated with lower hazard for recurrence and death. In Table 4, positive coefficients suggest that higher receptor levels are linked to longer time to event, reaffirming the protective biological effect, though the strength and significance vary slightly across models.
- Frailty Effects: The multiplicative frailty models in both tables adjust for unobserved heterogeneity. These models generally yield more conservative standard errors and sometimes reveal stronger covariate effects—particularly in Table 4—implying that accounting for latent patient‐level variation uncovers clearer relationships.

**TABLE 3:** Rotterdam Tumor Bank Data: Estimates (Est), standard errors (SE), and RR for the hazard‐ratio parameters, n=1546.

	Cox‐marginalized [39]			Cox without frailty I, II, III								
	Est (SE)	RR	p	Est (SE)	RR	p						
θ	2.52 (0.54)	—										
	Transition: surgery → relapse, 0→1, 974 events											
Age at surgery (/10)	−0.15 (0.06)	0.86	0.014	−0.16 (0.04)	0.85	0.000						
lymph nodes (log)	0.42 (0.04)	1.53	0.000	0.43 (0.04)	1.54	0.000						
estrogen (log)	−0.03 (0.02)	0.97	0.186	−0.04 (0.02)	0.96	0.027						
progesterone (log)	−0.04 (0.02)	0.96	0.065	−0.02 (0.02)	0.98	0.206						
Postmenopausal (vs. pre)	0.13 (0.13)	1.14	0.296	0.18 (0.12)	1.19	0.130						
Tumor size (ref <20 mm)												
20 − 50 mm	0.20 (0.07)	1.22	0.006	0.21 (0.08)	1.24	0.006						
>50 mm	0.38 (0.11)	1.46	0.001	0.43 (0.10)	1.54	0.000						
Hormone therapy	−0.38 (0.08)	0.68	0.000	−0.42 (0.09)	0.66	0.000						
Chemotherapy	−0.37 (0.11)	0.69	0.001	−0.47 (0.09)	0.63	0.000						
Tumor grade 3 (vs. 2)	0.21 (0.08)	1.23	0.008	0.24 (0.08)	1.27	0.003						

	Transition: surgery → death, 0→2, 106 events											
Age at surgery (/10)	1.32 (0.37)	3.74	0.000	1.36 (0.14)	3.88	0.000						
lymph nodes (log)	0.13 (0.12)	1.14	0.298	0.14 (0.12)	1.14	0.267						
estrogen (log)	−0.01 (0.06)	0.99	0.816	−0.05 (0.06)	0.95	0.436						
progesterone (log)	0.08 (0.06)	1.08	0.205	0.11 (0.06)	1.11	0.076						
Postmenopausal (vs. pre)	−0.30 (0.50)	0.74	0.554	−0.31 (0.63)	0.73	0.624						
Tumor size (ref. <20 mm)												
20–50 mm	−0.16 (0.25)	0.85	0.526	−0.10 (0.24)	0.91	0.682						
>50 mm	0.15 (0.31)	1.16	0.634	0.17 (0.30)	1.19	0.557						
Hormone therapy	−0.21 (0.25)	0.81	0.389	−0.27 (0.24)	0.76	0.262						
Chemotherapy	−0.22 (0.81)	0.81	0.789	−0.20 (0.55)	0.81	0.714						
Tumor grade 3 (vs. 2)	−0.01 (0.28)	0.99	0.961	0.01 (0.23)	1.01	0.978						

	Transition: relapse → death, $1\to 2^{\prime }$, 771 events out of 974 patients at risk											
	Cox‐marginalized [39]			Cox without frailty I			Cox without frailty II			Cox without frailty III		
	Est (SE)	RR	p	Est (SE)	RR	p	Est (SE)	RR	p	Est (SE)	RR	p
Age at relapse (/10)	0.03 (0.08)	1.03	0.700	0.06 (0.05)	1.07	0.202	0.12 (0.05)	1.13	0.017	0.12 (0.05)	1.13	0.017
lymph nodes (log)	0.25 (0.05)	1.28	0.000	0.08 (0.04)	1.09	0.061	0.08 (0.05)	1.08	0.091	0.07 (0.04)	1.07	0.132
estrogen (log)	−0.03 (0.02)	0.97	0.193	−0.02 (0.02)	0.98	0.414	−0.01 (0.02)	0.99	0.529	−0.01 (0.02)	0.99	0.682
progesterone (log)	−0.08 (0.02)	0.92	0.000	−0.11 (0.02)	0.89	0.000	−0.12 (0.02)	0.89	0.000	−0.12 (0.02)	0.88	0.000
Postmenopausal (pre)	−0.05 (0.13)	0.95	0.731	−0.04 (0.14)	0.96	0.782	−0.15 (0.14)	0.86	0.292	−0.15 (0.14)	0.86	0.282
Tumor size (ref. <20 mm)												
20–50 mm	0.23 (0.07)	1.26	0.001	0.25 (0.09)	1.28	0.009	0.24 (0.10)	1.27	0.012	0.24 (0.10)	1.27	0.012
>50 mm	0.40 (0.10)	1.49	0.000	0.30 (0.12)	1.35	0.009	0.26 (0.12)	1.29	0.026	0.27 (0.12)	1.31	0.022
Hormone therapy	−0.18 (0.09)	0.84	0.037	−0.01 (0.10)	0.99	0.951	0.04 (0.10)	1.04	0.672	0.07 (0.10)	1.07	0.519
Chemotherapy	−0.16 (0.13)	0.85	0.227	0.08 (0.11)	1.09	0.440	0.17 (0.11)	1.18	0.129	0.19 (0.11)	1.20	0.093
Tumor grade 3 (vs. 2)	0.21 (0.09)	1.23	0.024	0.13 (0.09)	1.14	0.167	0.12 (0.09)	1.28	0.200	0.14 (0.10)	1.15	0.133
SV	—	—	—	—	—	—	−0.31 (0.07)	0.73	0.000	1.40 (0.66)	4.19	0.033
Splines 20%	—	—	—	—	—	—	—	—	—	−2.89 (1.09)	0.06	0.008
Splines 40%	—	—	—	—	—	—	—	—	—	1.49 (0.88)	4.45	0.089
Splines 60%	—	—	—	—	—	—	—	—	—	−0.47 (0.59)	0.62	0.419
Splines 80%	—	—	—	—	—	—	—	—	—	0.18 (0.36)	1.20	0.617

**TABLE 4:** Rotterdam Tumor Bank Data: Estimates (Est), standard errors (SE) and p‐value, n=1546.

	AFT multiplicative [88]		AFT without frailty I, II, III					
	Est (SE)	p	Est (SE)	p				
θ	2.18 (0.73)	—						
	Transition: surgery → relapse, 0→1, 974 events							
Age at surgery (/10)	0.14 (0.06)	0.012	0.13 (0.06)	0.024				
lymph nodes (log)	−0.40 (0.05)	0.000	−0.41 (0.06)	0.000				
estrogen (log)	0.07 (0.03)	0.030	0.08 (0.03)	0.016				
progesterone (log)	0.09 (0.03)	0.000	0.08 (0.03)	0.006				
Postmenopausal (vs. pre)	−0.34 (0.15)	0.023	−0.30 (0.17)	0.075				
Tumor size (ref <20 mm)								
20‐50 mm	−0.32 (0.09)	0.001	−0.30 (0.09)	0.000				
>50 mm	−0.49 (0.11)	0.000	−0.48 (0.09)	0.000				
Hormone therapy	0.60 (0.13)	0.000	0.56 (0.14)	0.000				
Chemotherapy	0.49 (0.11)	0.000	0.47 (0.11)	0.000				
Tumor grade 3 (vs. 2)	−0.25 (0.09)	0.004	−0.25 (0.09)	0.004				

	Transition: surgery → death, 0→2, 106 events							
Age at surgery (/10)	−0.43 (0.14)	0.002	−0.99 (0.14)	0.000				
lymph nodes (log)	−0.14 (0.08)	0.091	0.06 (0.11)	0.568				
estrogen (log)	0.04 (0.04)	0.287	0.04 (0.05)	0.446				
progesterone (log)	0.01 (0.04)	0.827	−0.11 (0.05)	0.039				
Postmenopausal (vs. pre)	−0.15 (0.34)	0.647	0.21 (0.43)	0.628				
Tumor size (ref. <20 mm)								
20–50 mm	−0.13 (0.15)	0.376	0.06 (0.21)	0.753				
>50 mm	−0.19 (0.18)	0.275	−0.02 (0.22)	0.927				
Hormone therapy	0.41 (0.18)	0.019	−0.18 (0.27)	0.507				
Chemotherapy	1.13 (0.30)	0.000	0.31 (0.39)	0.431				
Tumor grade 3 (vs. 2)	−0.06 (0.13)	0.641	−0.13 (0.16)	0.426				

	Transition: relapse → death, $1\to 2^{\prime }$, 771 events out of 974 patients at risk							
	AFT multiplicative [88]		AFT without frailty I		AFT without frailty II		AFT without frailty III	
	Est (SE)	p	Est (SE)	p	Est (SE)	p	Est (SE)	p
Age at relapse (/10)	0.00 (0.07)	0.956	−0.12 (0.08)	0.141	−0.10 (0.06)	0.088	−0.10 (0.05)	0.049
lymph nodes (log)	−0.25 (0.07)	0.000	0.01 (0.09)	0.908	0.04 (0.06)	0.493	0.02 (0.05)	0.619
estrogen (log)	0.04 (0.05)	0.341	0.14 (0.07)	0.038	0.14 (0.03)	0.000	0.17 (0.03)	0.000
progesterone (log)	0.13 (0.04)	0.001	0.17 (0.05)	0.001	0.10 (0.04)	0.011	0.07 (0.04)	0.077
Postmenopausal (pre)	−0.21 (0.17)	0.203	−0.04 (0.25)	0.868	−0.13 (0.18)	0.466	−0.20 (0.14)	0.135
Tumor size (ref. <20 mm)								
20–50 mm	−0.37 (0.14)	0.008	−0.08 (0.16)	0.619	−0.24 (0.12)	0.049	−0.26 (0.13)	0.038
>50 mm	−0.52 (0.17)	0.002	−0.06 (0.21)	0.784	−0.22 (0.14)	0.115	−0.20 (0.14)	0.164
Hormone therapy	0.39 (0.14)	0.005	0.24 (0.17)	0.145	0.34 (0.10)	0.000	0.35 (0.13)	0.006
Chemotherapy	0.23 (0.18)	0.205	−0.11 (0.21)	0.608	−0.17 (0.12)	0.158	−0.13 (0.13)	0.328
Tumor grade 3 (vs. 2)	−0.26 (0.13)	0.047	−0.23 (0.13)	0.095	0.36 (0.08)	0.000	−0.32 (0.10)	0.002
SV	—	—	—	—	0.43 (0.10)	0.000	0.65 (0.55)	0.242
Splines 20%	—	—	—	—	—	—	0.23 (0.99)	0.818
Splines 40%	—	—	—	—	—	—	−1.11 (0.85)	0.193
Splines 60%	—	—	—	—	—	—	0.91 (0.51)	0.076
Splines 80%	—	—	—	—	—	—	−0.55 (0.66)	0.407

**FIGURE 5:**
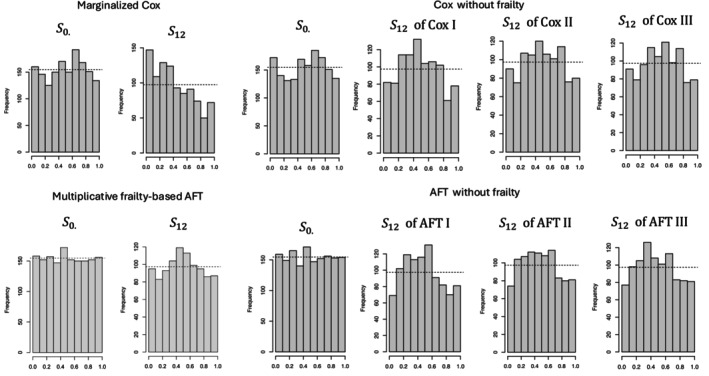
Rotterdam Tumor Bank Data: goodness of fit assessment based on randomized survival probabilities (RSPs). The plots display RSPs for remaining in State 0 ($S_{0}$) and for remaining in State 1 conditional on having experienced the non‐terminal event ($S_{12}$). Under a correctly specified model, the RSPs should follow a uniform distribution on (0,1).

To assess model fit, we used a visual goodness‐of‐fit (GOF) diagnostic based on randomized survival probabilities (RSPs) [88, 99]. Specifically, for illness–death models, two RSPs were evaluated: (i) the probability of remaining in State 0, denoted by $S_{0.}$; and (ii) the probability of remaining in State 1 among individuals who experienced the non‐terminal event, denoted by $S_{12}$. These RSPs are estimated for each observation given the observed covariates; for frailty‐based models, the marginal probabilities are obtained by integrating over the frailty distribution. Under a correctly specified model, these RSPs should follow a uniform distribution on (0,1). Model fit can therefore be visually assessed by comparing histograms of estimated RSPs to the standard uniform distribution. The resulting comparisons are shown in Figure 5. Among the eight models considered, the multiplicative frailty‐based AFT model appears to provide the best overall fit to the data. In summary, although the Cox and AFT models yield consistent directional interpretations for most covariates, the AFT framework offers a time‐based understanding of covariate effects, which can be more intuitive in clinical contexts. Moreover, the frailty‐adjusted models appear to better capture the complexity and heterogeneity in this dataset, enhancing both fit and interpretability.

This analysis highlights the complexity of modeling illness–death data. Frailty‐based approaches effectively capture unobserved dependence between time to non‐terminal and terminal events but require pre‐specifying the frailty distribution. In contrast, non‐frailty models avoid such distributional assumptions but necessitate careful modeling of the functional relationship between the non‐terminal event time and the transition $1\to 2^{\prime }$.

### 5.2 Between‐Subject Dependence

There has been limited exploration of illness‐death models in the context of clustered data, such as family or twin studies. Frailty‐based models and estimation methods for competing risks (i.e., Figure 1f) in clustered failure‐time data have been developed [77, 100, 101, 102]. However, extending these approaches to more complex settings of multistate models is challenging, primarily due to the varying strength of dependence between two cluster members across different transitions. Lee and Cook [92] developed an illness‐death model using the latent variable formulation of the competing‐risk model for the first transition, 0→1 or 0→2, and a copula model is adopted to accommodate dependence within clusters in the (possibly latent) times to transition from state 0 to 1.

## 6 Software Availability

This section offers an overview of the software and packages (in either R or Python) accessible for analyzing multistate models, highlighting the distinctive contributions of each package.

### 6.1 R Packages survival and mstate

The survfit function [32] of the package survival [103, 104] calculates and plots the Aalen‐Johansen estimators of cumulative incident functions within any multistate scenario. In particular, subjects can visit multiple states during the course of a study, subjects can start after time 0 (i.e., delayed entry), and they can start in any of the states. The standard error of the Aalen‐Johansen estimates is computed using an infinitesimal jackknife. The coxph function offers Cox regression analysis for each transition in a multistate model, with or without shared coefficients. The mstate package [105, 106] incorporates utilities for data preparation, descriptive analyses, hazard functions estimation, prediction using Aalen‐Johansen estimator and Cox regression modeling. Due to its modular approach, different models, like for instance additive hazards models, can be fitted for the transition intensities, while still allowing prediction based on Aalen‐Johansen. Also, functions for testing the Markov assumption are provided [107].

### 6.2 R package msm

The msm package [23] offers a suite of functions for simulating and analyzing continuous‐time Markov processes with piecewise‐constant intensity functions, as well as hidden Markov models that include unobserved (hidden) states; the latter are not discussed here. A unique feature of this package is that it facilitates proportional intensity regression analyses for Markov processes with piecewise‐constant baseline intensities when processes are subject to right‐censoring or under intermittent observation. The package is therefore well‐suited for modeling the type of data described in Section 4, as demonstrated in the example in Section 4.1. The package provides estimates for transition intensities, transition probability matrices, and expected time spent in each state. It also yields predictions for future state occupancy. Parameters are estimated via maximum likelihood, enabling standard inferential techniques for confidence intervals and hypothesis testing.

### 6.3 Python Package PyMSM

PyMSM [108] is a package for fitting competing risks and multistate models, offering flexible model specification, individual and population‐level predictions, and comprehensive statistical summaries and visualizations. Key features include: (1) Multistate Regression Model Fitting: Supports various survival analysis techniques, such as Cox regression, random survival forests [109], or user‐defined machine learning models. (2) Prediction via Monte Carlo Simulation: Using a fitted multistate model, PyMSM generates sample paths through Monte Carlo simulations. Given covariates, initial states, and times, it sequentially samples subsequent states and durations in each state based on the estimated model, ending when a terminal state is reached or a predefined maximum number of transitions is exceeded. Summary statistics, such as state occupancy probabilities and median state durations, are available after sampling multiple paths per observation. (3) Predefined Models and Data Simulation: Allows loading or configuring predefined multistate models and generating simulated survival data via random paths, providing a valuable tool for research.

### 6.4 R Package SmoothHazard

The SmoothHazard package [110] is designed for fitting regression models to interval‐censored data within illness‐death models. It includes algorithms for concurrently fitting regression models to the three transition intensities of an illness‐death model, where the transition times to State 1 (see Figure 1c) may be interval‐censored, and all event times can be right‐censored. The three baseline transition intensity functions are modeled either by Weibull distributions or, alternatively, by M‐splines in a semi‐parametric framework. Given specific covariates, the estimated transition intensities can be combined to produce estimates of cumulative incidence functions and life expectancies.

### 6.5 R Packages pseudo and eventglm

The pseudo R package [111] includes functions for computing pseudo‐values (see Section 2.3) for various marginal parameters of interest such as the cumulative incidence function, or the restricted mean time in a state. The R package eventglm also applies the pseudo‐values framework and includes plotting of residuals, the use of sampling weights, and corrected variance estimation.

### 6.6 R Package simMSM

The R package simMSM [112] simulates event histories for multistate models. It enables the generation of event histories featuring potentially non‐linear baseline hazard functions, as well as nonlinear time‐dependent or time‐independent covariates' effect, while also accounting for dependencies on past history. The random generation of event histories is achieved through inversion sampling applied to cumulative all‐cause hazard rate functions.

### 6.7 R Packages Targeting Illness‐Death Models With Within‐Subject Random Effects

The R package SemiCompRisks [113] uses Bayesian estimation techniques for frailty‐based illness‐death models, encompassing both conditional and additive models as delineated in Sections 5.1.1, 5.1.2. This package offers Cox‐type and accelerated failure time models incorporating gamma and normal frailty distributions, respectively. The frailty‐LTRC package (available at https://github.com/nirkeret/frailty‐LTRC) utilizes a pseudo‐likelihood approach for marginalized Cox models outlined in Section 5.1.1, using a gamma frailty. On the other hand, semicompAFT (available at https://github.com/leakats/semicompaft) implements a semi‐parametric AFT model under the multiplicative frailty setting discussed in Section 5.1.2, also using a gamma frailty distribution. frailtypack [114] is a package focusing particularly on handling frailty models and is versatile for time‐to‐event data in complex scenarios, including multistate models with recurrent events or competing risks.

## 7 Pros and Cons to Multistate Modeling

The intensity‐based framework for modeling multistate processes is well‐aligned with how information unfolds and is revealed over time. In particular, it recognizes that the past (history) influences the future, individuals are simultaneously at risk of more than one type of event, and that processes can terminate for many different reasons. Through the incorporation of internal and external time‐varying covariates they can offer useful insights into the association between dynamic factors and the disease progression or death.

The intensity‐based framework for examining how the occurrence of one type of event can alter the risk of another type of event is the basis for local dependence modeling [27]. In Section 2.2.1 we mentioned how the local dependence between disease recurrence and death can be studied in the framework of an illness‐death model. More generally joint models can be formed to study the co‐occurrence of disease (i.e., co‐morbidities), or the relationship between recurrent and terminal events—Cook et al. [115] use this framework to study the relation between recurrent skeletal events and death in trials of patients with bone metastases. This can be viewed as an extension of the illness‐death process where a countable number of non‐fatal events may occur. In this setting, the Aalen‐Johansen estimator [27] of transition probability matrix can be used to jointly estimate the restricted mean lifetime number of events and survival probabilities.

In non‐dynamic settings, association between two variables is typically thought of as a symmetric feature, in the sense that if X is dependent on Y, then Y is dependent on X. Intensity‐based models accommodate asymmetric relationships wherein the risk of one event may be altered by the occurrence of a second event, but the risk of the second type of event does not change upon the occurrence of the first type of event. This has natural appeal when one event is death and the dependence is necessarily asymmetric. Aalen [116] pointed out that local dependence modeling can yield insights into causal effects within the Granger school.

Although comprehensive multistate models offer advantages for capturing complex event histories, they are not widely adopted in practice. This hesitation may stem from limited familiarity with the methods among applied researchers, as well as concerns that these models require more elaborate modeling assumptions. Such complexity can raise concern about robustness, particularly when data are sparse or model assumptions are difficult to validate. In cancer clinical trials, times of interest include times to cancer progression, progression‐free death, and death following cancer progression. As illustrated in Section 2.2.1 these event times are naturally jointly modeled with a three or four state illness‐death process; see Figure 1c and d respectively. It is more common however, to assess treatment effects on the basis of progression‐free survival time, a composite endpoint defined as the time spent in state 0. While avoiding a competing risks problem and enabling one to carry out a simple time to event analysis, the regression coefficient of the treatment indicator does not yield an estimand with a clear interpretation [117]. Recent work on estimands motivated by the release of the ICH‐E9 Addendum aims to define clearly interpretable estimands of treatment effects in such settings and multistate models can play an important role in obtaining estimates for the expected time spent in different states [118], the probability of different paths, or for incorporating the introduction of co‐interventions or treatment switches [119]. Synthesis of summary statistics can then be carried out by assigning of relative values of different states [120] or by more explicit specification of utilities [121].

In any application one must weigh the desire to formulate comprehensive models which address the complexities of an underlying process, with the desire for simple interpretable estimands and robustness. There may be a clear understanding that processes are complex but if data are limited then complex models may be challenging to fit. This arose in Section 4.1 where we discussed the multistate analysis of joint damage in psoriatic arthritis but individuals were only under intermittent observation.

To summarize, multistate models offer several advantages: (i) they provide a comprehensive framework for representing complex processes; (ii) enable modeling of dependencies between events; (iii) support prediction for multiple types of outcomes; and (iv) many diagnostic tools from univariate survival analysis can be extended to this setting. However, these models also come with limitations: (i) it can be difficult to specify the correct form of history dependence; (ii) relevant time scales are sometimes unclear; (iii) intensity‐based modeling requires more assumptions than those typically made in simpler models; and (iv) reliable estimation demands sufficient events per transition, complicating sample size calculations compared to single‐event survival analysis.

While the focus of this paper is on statistical modeling and prediction of multistate processes, it is important to note that the regression models and methods discussed in this paper do not yield estimators with a causal interpretation of transition dynamics. Defining causal estimands for multistate processes—particularly in the presence of time‐dependent covariates, intermediate events, and feedback between states—requires the specification of potential outcomes and assumptions on treatment assignment mechanisms. Such causal estimands are often most naturally expressed in terms of marginal attributes, for example state occupation probabilities or functionals based on state occupancy over time, rather than transition‐specific intensities. In this context, methods based on pseudo‐values hold promise for causal analysis of multistate data. Recent work has begun to develop causal frameworks for multistate and event‐history data along these lines [119, 122, 123, 124, 125], but a comprehensive treatment of causal inference in multistate models lies beyond the scope of the present review and remains an active area of ongoing research.

## 8 Discussion

In this paper, we have presented various approaches to modeling multistate processes, with a focus on both intensity‐based and marginal models. Sections 2 and 3 lay out the foundational concepts for understanding these models and their practical utility. Section 4 builds on this by addressing the challenges posed by intermittent observation in continuous‐time processes, which is a frequent issue in clinical studies where transitions between states are not continuously observed. Our exploration of frailty‐based models in Section 5 illustrates their ability to capture unobserved heterogeneity. The approaches discussed in this paper provide critical tools for handling the complexities of real‐world data, enabling researchers to make more accurate inferences about the dynamics of processes such as disease progression.

While the frailty‐based models provide a useful framework for addressing subject‐specific unobserved covariates, they also introduce additional complexities, especially regarding the assumptions about dependence structures. Future research should focus on extending these methods to accommodate more complex multistate processes.

Intensity‐based regression models do not yield estimates of the model parameters that are robust to the omission of important covariates. In causal parlance, conditioning on occupancy of the recurrence state creates a collider bias; see Section 8.4 of Cook and Lawless [16]. With time‐fixed covariates, an alternative way of assessing their relation to the multistate process is through stratification and computation of expected sojourn times in different states for each stratum.

Validating multistate models presents significant challenges, particularly when evaluating their predictive performance or assessing goodness‐of‐fit with real‐world data. A comprehensive literature review of existing model checking methods, along with an identification of the key unresolved issues, would be highly valuable for advancing this area.

Another important area not covered here is the application of machine learning methods to multistate survival data. While methods for application of machine learning algorithms to multistate survival analysis is still emerging, it shows considerable potential for improving prediction accuracy, particularly in healthcare settings [126, 127, 128]. A key challenge is balancing the predictive performance of machine learning with the need for interpretability and robustness, both crucial for clinical decision‐making. Modern survival‐based machine learning methods also offer tools for interpretation, including variable importance measures and state‐specific risk summaries [129]. Another major challenge is uncertainty quantification for advanced machine learning methods, such as deep neural networks. A recent work [130] proposed a method for quantifying uncertainty in survival analysis which can accommodate deep learning approaches. Machine learning algorithms can be directly applied to multistate processes through the pseudo‐value approach discussed in Section 3.3, as these algorithms are typically designed for cross‐sectional rather than dynamic data. When a relevant time horizon can be specified, machine learning can be used to predict state occupancy probabilities. Early work in this direction has focused on competing and semi‐competing risks processes [131]. As the field advances, the integration of machine learning into multistate modeling frameworks will likely open new avenues for analyzing complex survival data.

## Funding

This work was supported by the Israel Science Foundation (Grant No. 767/21), the Tel Aviv University Center for AI and Data Science (TAD), the Discovery Grant from the Natural Sciences and Engineering Research Council of Canada (Grant No. RGPIN‐2017‐04207), and the Canadian Institutes of Health Research (Grant No. FRN 13887). M.A. is a Distinguished James McGill Professor of Biostatistics at McGill University. The work of M.P.P. was supported by funding received from Slovenian Research and Innovation Agency (grant P3‐0154).

## Conflicts of Interest

The authors declare no conflicts of interest.

## Supporting information




**Data S1**: Supporting Information.

## Data Availability

The data that support the findings of this study are available in CRAN Packages at https://github.com/therneau/survival. These data were derived from the following resources available in the public domain: – survival R package, https://github.com/therneau/survival.
