# Supplementary material for: An Overview and Recent Developments in the Analysis of Multistate Processes
Source: Stat Med. 2026 May 14;45:e70493. doi: 10.1002/sim.70493 (PMC13174117; doi:10.1002/sim.70493)
Supplement: Supplementary file 1 — Data S1: Supporting Information. [file SIM-45-0-s001.pdf]

Supplementary Material
An Overview and Recent Developments in the Analysis of Multistate Processes

Malka Gorfine, Richard J. Cook, Per Kragh Andersen, Terry M. Therneau, Pierre Joly, Hein Putter, Maja Pohar Perme, Michal Abrahamowicz

On Behalf of Topic Group 8 "Survival Analysis" of the STRATOS initiation.

Contents

S1 Code of Section 2.2.1 - The Colon Cancer Study Revisited, I **2**

S2 Code of Section 2.4.1 - The Colon Cancer Study Revisited, II **4**

S3 Code of Section 4.1 - The Psoriatic Arthritis Data Revisited **6**

S4 Codes of Section 5.1.3 - The Rotterdam Tumor Bank Data Revisited. **8**
S4.1 Conditional frailty with Cox-type model . . . 8
S4.2 The AFT additive-frailty model . . . 11

S5 Glossary of Notation **13**

S1 Code of Section 2.2.1 - The Colon Cancer Study Revisited, I

```
palette("Okabe-Ito")

c1 <- subset(colon, etype== 1) # recurrence
c2 <- subset(colon, etype== 2) # death
# There are 5 subjects with recurrence coded on the same day as death.
# Make them recurrent one day earlier than their death (avoid 0 length intervals)
tied.id <- (c1$time == c2$time & c1$status==1 & c2$status==1)
c1$time[tied.id] <- c1$time[tied.id] -1

# Create the counting process dataset
# The variable time1 indicates the start of each interval time2 marks its end.
# The variable state denotes the state entered at the end of the corresponding interval.
cdata <- tmerge(subset(c2, ,-c(study, time, status, etype)),
                c2, id=id, death= event(time, status),
                options=list(tstart="time1", tstop="time2"))
cdata <- tmerge(cdata, c1, id= id, recur= event(time, status),
                td.recur = tdc(time))

> head(cdata[,c(1:4,13:16,18:21)],5)
  id      rx sex age    time1    time2 death recur                 state age10 male trt
1  1 Lev+5FU   1  43 0.000000 2.650240     0     1                 recur   4.3    1   1
2  1 Lev+5FU   1  43 2.650240 4.164271     1     0 death post-recurrence   4.3    1   1
3  2 Lev+5FU   1  63 0.000000 8.451745     0     0                censor   6.3    1   1
4  3     Obs   0  71 0.000000 1.483915     0     1                 recur   7.1    0   0
5  3     Obs   0  71 1.483915 2.636550     1     0 death post-recurrence   7.1    0   0

# Make the state variable with recurrence and death pre and post recurrence
cdata$state <- with(cdata, factor(recur+ 2*death + 3*td.recur, c(0,1,2,5,3),
                                  c("censor", "recur", "death pre-recurrence",
                                    "death post-recurrence","censor")))

> table(cdata$state)

              censor                recur  death pre-recurrence death post-recurrence
                 475                  468                    38                   414

cdata$trt <- 1*(cdata$rx == "Lev+5FU")
# a 0/1 treatment variable, 1=Lev+5FU, 0= Observation or Lev
# Output in years gives nicer plot axes
cdata$time1 <- cdata$time1/365.25
cdata$time2 <- cdata$time2/365.25
# Check for consistency
check <- survcheck(Surv(time1, time2, state) ~ 1, cdata, id=id)
check


#Nelson-Aalen (NA) estimator of cumulative transition intensities
na0 <- survfit(Surv(time1, time2, state) ~ 1, cdata, id=id, stype=1, ctype=1) # overall
na1 <- survfit(Surv(time1, time2, state) ~ trt, cdata, id=id, stype=1, ctype=1) # by arm

# Extract cumulative hazard data from na1 with summary
na1_summary <- summary(na1, cumhaz = TRUE)

na1_df <- data.frame(
  time = na1_summary$time,
  strata = factor(na1_summary$strata, labels = c("Control", "5FU+Lev")),
  cumhaz = na1_summary$cumhaz[,1],       # Only the first transition
  std_err = na1_summary$std.chaz[,1]      # Only the standard error for the first transition
)
```

```r
# Add confidence intervals for the first transition
na1_df <- na1_df %>%
  mutate(
    lower = pmax(0, cumhaz - 1.96 * std_err),  # 95% confidence interval lower bound
    upper = cumhaz + 1.96 * std_err            # 95% confidence interval upper bound
  )

# Plot2(a)
ggplot(na1_df, aes(x = time, y = cumhaz, color = strata, fill = strata)) +
  geom_line(size = 1) + # Plot the cumulative hazard curve
  geom_ribbon(aes(ymin = lower, ymax = upper), alpha = 0.1) +  # Add shaded area
  labs(x = "Years Since Enrollment", y = expression('NA Estimate of '*Lambda[0][1](t))) +
  scale_color_manual(values = c("5FU+Lev" = "red", "Control" = "blue")) +
  scale_fill_manual(values = c("5FU+Lev" = "red", "Control" = "blue")) +
  theme_minimal() +
  theme(
    axis.title.x = element_text(size = 16),  # Change size for x-axis title
    axis.title.y = element_text(size = 16),
    legend.title = element_blank(),      # Remove legend title for simplicity
    legend.position = c(0.1, 0.9),        # Position the legend in the upper left corner
    panel.border = element_rect(color = "black", fill = NA, size = 0.4),
    panel.grid = element_blank()
      ) +
  guides(color = guide_legend(override.aes = list(size = 2)))  # Adjust legend line width

#plot2(b)
na1_df <- data.frame(
  time = na1_summary$time,
  strata = factor(na1_summary$strata, labels = c("Control", "5FU+Lev")),
  cumhaz_1_2 = na1_summary$cumhaz[,2],
  std_err_1_2 = na1_summary$std.chaz[,2],
  cumhaz_2_3 = na1_summary$cumhaz[,3],
  std_err_2_3 = na1_summary$std.chaz[,3]
)

na1_long <- na1_df %>%
  pivot_longer(
    cols = starts_with("cumhaz"),
    names_to = "transition",
    values_to = "cumhaz"
  ) %>%
  mutate(
    std_err = case_when(
      transition == "cumhaz_1_2" ~ std_err_1_2,
      transition == "cumhaz_2_3" ~ std_err_2_3
    ),
    lower =  pmax(0, cumhaz - 1.96 * std_err),  # Calculate lower CI
    upper = cumhaz + 1.96 * std_err     # Calculate upper CI
  )

ggplot(na1_long, aes(x = time, y = cumhaz, color = strata, fill = strata)) +
  geom_line(size = 1, aes(linetype = transition, group = interaction(strata, transition))) +
  geom_ribbon(aes(ymin = lower, ymax = upper,
    group = interaction(strata, transition)), alpha = 0.1) +
  labs(x = "Years Since Enrollment", y = expression('NA Estimate of '*Lambda[j][2](t))) +

  # Set custom colors and fills for strata
  scale_color_manual(
    values = c("5FU+Lev" = "red", "Control" = "blue"),
    labels = c("5FU+Lev" = "5FU+Lev", "Control" = "Control")
```

```
  ) +
  scale_fill_manual(values = c("5FU+Lev" = "red", "Control" = "blue")) +

  # Define custom linetype labels using TeX for mathematical notation
  scale_linetype_manual(
    values = c("solid", "dashed"),
    labels = c(TeX("$\\Lambda_{\\0\\2}$"), TeX("$\\Lambda_{12\\'}$"))
  ) +
  # Adjust theme and legend
  theme_minimal() +
  theme(
    axis.title.x = element_text(size = 16),  # Change size for x-axis title
    axis.title.y = element_text(size = 16),
    legend.title = element_blank(),           # Remove legend title
    legend.position = c(0.1, 0.9),            # Position legend in the upper left corner
    legend.text = element_text(size = 14),
    panel.border = element_rect(color = "black", fill = NA, size = 0.4),
    panel.grid = element_blank()
      ) +
  # Override aesthetics for clearer legend display
  guides(
    color = guide_legend(override.aes = list(size = 1.2)),
    linetype = guide_legend(override.aes = list(size = 1.2))
  )


# Plots 2(c) and 2(d)
#AJ for CIF
plot(na1$time[1:795],(na1$pstate[,"recur"]+na1$pstate[,"death post-recurrence"])[1:795],
     type = 's',
     lwd=2, lty=1, col=c("blue"),
     xlab="Years since enrollment", ylab=expression('AJ Estimate of '*F[1](t)))
lines(na1$time[796:1193],(na1$pstate[,"recur"]+na1$pstate[,"death post-recurrence"])[796:1193],
     type = 's',
     lwd=2, lty=1, col=c("red"))
legend(-0.3, 0.6, c("5FU+Lev", "Control"),
       lwd=2, lty=1, col=c("red","blue"), bty='n')

plot(na1, noplot=c("(s0)","recur"),
     lwd=2, lty=c(1,1,2,2), col=c("blue","red", "blue","red"),
     xlab="Years since enrollment",
     ylab=expression('AJ Estimate of '*F[2](t)* ' and ' *F[2*"'"](t)))
legend(-0.2, 0.55, c(expression(F[2](t)*" 5FU+Lev"),
                     expression(F[2*"'"](t)*' 5FU+Lev'),
                     expression(F[2](t)*' Control'),
                     expression(F[2*"'"](t)*' Control')),
       lwd=2, lty=1:2, col=c("red", "red", "blue", "blue"), bty='n')
```

S2 Code of Section 2.4.1 - The Colon Cancer Study Revisited, II

```
> table(cdata$extent)
  1    2    3    4
 26  140 1157   72

> table(cdata$extent,cdata$state)
    censor recur death pre-recurrence death post-recurrence
  1     17     5                    1                     3
  2     70    34                    5                    31
```

```
  3    374   400                    28                    355
  4     14    29                     4                     25

  > cfit2New <- coxph(Surv(time1, time2, state) ~ trt + extent01 + node4
+                    ,data= cdata, id=id, robust = FALSE)
> cfit2New
Call:
coxph(formula = Surv(time1, time2, state) ~ trt + extent01 +
    node4, data = cdata, robust = FALSE, id = id)


1:2            coef exp(coef) se(coef)      z        p
  trt      -0.50584   0.60300  0.10628 -4.759 1.94e-06
  extent01  0.64909   1.91380  0.16803  3.863 0.000112
  node4     0.84501   2.32799  0.09594  8.807  < 2e-16


1:3          coef exp(coef) se(coef)     z     p
  trt      0.0346    1.0352   0.3331 0.104 0.917
  extent01 0.1084    1.1145   0.4488 0.242 0.809
  node4    0.4864    1.6265   0.3733 1.303 0.193


2:4          coef exp(coef) se(coef)     z        p
  trt      0.2346    1.2645   0.1126 2.083 0.037231
  extent01 0.3040    1.3552   0.1796 1.692 0.090642
  node4    0.3792    1.4610   0.1031 3.678 0.000235

 States:  1= (s0), 2= recur, 3= death pre-recurrence, 4= death post-recurrence

Likelihood ratio test=143.7  on 9 df, p=< 2.2e-16
n= 1395, unique id= 929, number of events= 920
> # 95% confidence intervals of HR
> temp <- summary(cfit2New)$conf.int[ , c(1,3,4)]
> temp
             exp(coef) lower .95 upper .95
trt_1:2      0.6029969 0.4896068 0.7426475
extent01_1:2 1.9137966 1.3767796 2.6602787
node4_1:2    2.3279944 1.9289178 2.8096367
trt_1:3      1.0352090 0.5388494 1.9887888
extent01_1:3 1.1144967 0.4624284 2.6860437
node4_1:3    1.6264570 0.7824650 3.3808057
trt_2:4      1.2644642 1.0139800 1.5768257
extent01_2:4 1.3552118 0.9530066 1.9271629
node4_2:4    1.4610437 1.1937605 1.7881717

# generate the forest plot - Figure 3
opar <- par(mar=c(5,7, 1, 1))  # more room for y axis labels
yy <- c(outer(3:1, c(8, 0, 4), "+"))
plot(c(.5,4), range(yy), log='x',  type="n",
     xlab="Hazard Ratio", ylab="", yaxt= 'n')
axis(2, at=yy, label=rep(c("Lev+5FU", "Extent 3 or 4", "Nodes > 4") ,3),
     las=2)

points(temp[,1], yy, pch=19)
segments(temp[,2], yy, temp[,3], yy, lwd=1.5)

abline(h=c(4,8), lwd=2, lty=2, col="red")
abline(v=1, lty=3)
text(c(2.5, 2.5, 2.5), c(11, 6.5, 3.5), c("Entry to Recurrence", "Recurrence to Death",
                                     "Entry to Death"))
```

```
par(opar)
```

S3 Code of Section 4.1 - The Psoriatic Arthritis Data Revisited

```
baseline_df <- psor %>%
  group_by(ptnum) %>%
  filter(months == min(months)) %>%
  select(ptnum, hieffusn_baseline = hieffusn, ollwsdrt_baseline = ollwsdrt)

baseline_df$ollwsdrt_baseline <- ifelse(baseline_df$ollwsdrt_baseline==0,1,0)

merged_df <- psor %>%
  left_join(baseline_df, by = "ptnum")

cutpoints <- c(-Inf, 5, 10, 20, Inf)

# Create a new categorical variable for the intervals
merged_df <- merged_df %>%
  mutate(
    time_interval = cut(months, breaks = cutpoints,
                        labels = c("1", "2", "3", "4"),
                        include.lowest = TRUE)
  )

> head(merged_df[,c(1:3,6:8)],9)
  ptnum  months state hieffusn_baseline ollwsdrt_baseline time_interval
1     1  6.4606     1                 0                 0             2
2     1 17.0780     1                 0                 0             3
3     2 26.3217     1                 0                 1             4
4     2 29.4839     3                 0                 1             4
5     2 30.5763     4                 0                 1             4
6     5  0.0753     1                 0                 1             1
7     5 12.8939     1                 0                 1             3
8     5 13.3730     2                 0                 1             3
9     5 14.3504     2                 0                 1             3

psor.q <- rbind(c(0,0.1,0,0),
                c(0,0,0.1,0),
                c(0,0,0,0.1),
                c(0,0,0,0))
psorpci.msm <- msm(state ~ months, subject = ptnum,
                   data = merged_df,
                   qmatrix = psor.q,
                   covariates = ~ hieffusn_baseline + ollwsdrt_baseline,
                   gen.inits=TRUE,
                   pci = c(5,10, 20),
                   control = list(REPORT=1,trace=2))
HR = hazard.msm(psorpci.msm)
> HR
$hieffusn_baseline
                         HR         L        U
State 1 - State 2 2.100252 0.9595722 4.596899
State 2 - State 3 1.709818 0.9548091 3.061846
State 3 - State 4 1.358133 0.7385758 2.497407

$ollwsdrt_baseline
                          HR         L        U
State 1 - State 2 1.2704387 0.7372401 2.189266
```

```
State 2 - State 3 2.1677660 1.2500937 3.759086
State 3 - State 4 0.6981905 0.3422453 1.424329

$`timeperiod[5,10)`
                         HR         L        U
State 1 - State 2 0.7181648 0.3868209 1.333332
State 2 - State 3 0.8283696 0.3936377 1.743218
State 3 - State 4 1.3251053 0.4340788 4.045128

$`timeperiod[10,20)`
                         HR         L         U
State 1 - State 2 0.4195343 0.1874067 0.9391822
State 2 - State 3 0.7961147 0.4064397 1.5593917
State 3 - State 4 1.1737629 0.3810700 3.6153969

$`timeperiod[20,Inf)`
                        HR         L        U
State 1 - State 2 1.565952 0.7626200 3.215501
State 2 - State 3 1.126529 0.5017957 2.529052
State 3 - State 4 1.372552 0.4309212 4.371793


t = seq(0, 60, by = 0.1)
p02 = c(); p03 = c(); p04 = c()
for(i in t){
  p = pmatrix.msm(psorpci.msm, t=i)
  p02 = append(p02, p[1,2])
  p03 = append(p03, p[1,3])
  p04 = append(p04, p[1,4])
}

plot(t, p04, type="l", lwd = 2, col = "blue", xlab = "Time from Diagnosis",
     ylab = "Transition Probability")
lines(t, p03, lwd = 2, col = "green")
lines(t, p02, lwd = 2, col = "red")
legend(-0.5, 1, c(expression(P[0][1](t)),
                  expression(P[0][2](t)),
                  expression(P[0][3](t))),
       lwd=2, lty=1, col=c("red", "green", "blue"), bty='n')


# Figure 4(a)
first5 = psor[psor$ptnum %in% unique(psor$ptnum)[1:5], 1:3]

plot(0, ylim = c(0.7, 5.3), xlim = c(0, 32), xaxt = "n", yaxt = "n", ylab = "Patient ID",
     xlab = "Years Since Disease Onset", tck = 0, las = 1)
axis(1, at = seq(0, 32, 5))

patient_id = unique(first5$ptnum)

# Create a mapping from patient index to letters
patient_labels = c("A", "B",  "C", "D", "E")

j = 0
for (i in patient_id) {
  id_data = first5[first5$ptnum == i,]
  j = j + 1
  lines(c(id_data$months[1], tail(id_data$months, n=1)),
        c(j, j), lty = 1)
  points(id_data$months, rep(j, length(id_data$months)),
         pch = rep("|", length(id_data$months)))
```

```
}

# Replace y-axis numbers with letters
axis(2, at = 1:5, labels = patient_labels, las = 2)


# Figure 4(b)
# TransProb
t = seq(0, 30, by = 0.1)
p02 = c()
p03 = c()
p04 = c()
for(i in t){
  p = pmatrix.msm(psorpci.msm, t=i)
  p02 = append(p02, p[1,2])
  p03 = append(p03, p[1,3])
  p04 = append(p04, p[1,4])
}

plot(t, p04, type="l", lwd = 2, col = "blue", xlab = "Years Since Disease Onset",
     ylab = "Transition Probability")
lines(t, p03, lwd = 2, col = "green")
lines(t, p02, lwd = 2, col = "red")
legend(-0.5, 0.75, c(expression(P[0][1](t)),
                  expression(P[0][2](t)),
                  expression(P[0][3](t))),
       lwd=2, lty=1, col=c("red", "green", "blue"), bty='n')
```

S4 Codes of Section 5.1.3 - The Rotterdam Tumor Bank Data Revisited.

All the codes used for Table 3 are available at https://github.com/LeaKats/semicompAFT. Here we provide also the two codes based on the package `SemiCompRisks`. The codes of the marginalized Cox and multiplicative AFT approach are too long to be included here and can be found in the above GitHub address.

S4.1 Conditional frailty with Cox-type model

```
#Load packages
suppressPackageStartupMessages(library(SemiCompRisks))
suppressPackageStartupMessages(library(survival))
suppressPackageStartupMessages(library(readxl))

###### Load data ######
data <- as.data.frame(read_excel("breast.xlsx"))
data<-data[data$nodes>0,]

data$size20_50<-ifelse(data$size=="20-50",1,0)
data$size50<-ifelse(data$size==">50",1,0)
data$grade3<-ifelse(data$grade==3,1,0)
data$log_pgr<-log(1+data$pgr)
data$log_er<-log(1+data$er)
data$age_10<-data$age/10
data$log_nodes<-log(data$nodes)

n<-dim(data)[1]

data$rtime_years<-data$rtime/365.25
data$rtime_10<-data$rtime/10
data$rtime_years_10<-data$rtime/365.25/10
data$dtime_10<-data$dtime/10
```

```
data$dtime_years<-data$dtime/365.25
#dealing with cases when recur time=death time (added 0.5 day):
data$dtime_years[(data$recur==1)&(data$rtime==data$dtime)]<-
  data$dtime_years[(data$recur==1)&(data$rtime==data$dtime)]+0.5/365.25
data$age_at_relapse_10<-data$age_10+data$rtime_years_10

set.seed(1)

form <- Formula(rtime + recur | dtime + death ~
            age_10+log_nodes+log_er+log_pgr+meno+size20_50+size50+hormon+chemo+grade3 |
            age_10+log_nodes+log_er+log_pgr+meno+size20_50+size50+hormon+chemo+grade3 |
            age_at_relapse_10+log_nodes+log_er+log_pgr+meno+size20_50+size50+hormon+chemo+grade3)

#####################
## Hyperparameters ##
#####################

## Subject-specific frailty variance component
##  - prior parameters for 1/theta
##
## Subject-specific frailty variance component
##  - prior parameters for 1/theta
##
theta.ab <- c(0.7, 0.7)

## Weibull baseline hazard function: alphas, kappas
##
WB.ab1 <- c(0.5, 0.01) # prior parameters for alpha1
WB.ab2 <- c(0.5, 0.01) # prior parameters for alpha2
WB.ab3 <- c(0.5, 0.01) # prior parameters for alpha3
##
WB.cd1 <- c(0.5, 0.05) # prior parameters for kappa1
WB.cd2 <- c(0.5, 0.05) # prior parameters for kappa2
WB.cd3 <- c(0.5, 0.05) # prior parameters for kappa3

## PEM baseline hazard function
##
PEM.ab1 <- c(0.7, 0.7) # prior parameters for 1/sigma_1^2
PEM.ab2 <- c(0.7, 0.7) # prior parameters for 1/sigma_2^2
PEM.ab3 <- c(0.7, 0.7) # prior parameters for 1/sigma_3^2
##
PEM.alpha1 <- 10 # prior parameters for K1
PEM.alpha2 <- 10 # prior parameters for K2
PEM.alpha3 <- 10 # prior parameters for K3

## MVN cluster-specific random effects
##
Psi_v <- diag(1, 3)
rho_v <- 100

## DPM cluster-specific random effects
##
Psi0  <- diag(1, 3)
rho0  <- 10
aTau  <- 1.5
bTau  <- 0.0125

##
hyperParams <- list(theta=theta.ab,
                    WB=list(WB.ab1=WB.ab1, WB.ab2=WB.ab2, WB.ab3=WB.ab3,
                            WB.cd1=WB.cd1, WB.cd2=WB.cd2, WB.cd3=WB.cd3),
```

```
                    PEM=list(PEM.ab1=PEM.ab1, PEM.ab2=PEM.ab2, PEM.ab3=PEM.ab3,
                             PEM.alpha1=PEM.alpha1, PEM.alpha2=PEM.alpha2, PEM.alpha3=PEM.alpha3),
                    MVN=list(Psi_v=Psi_v, rho_v=rho_v),
                    DPM=list(Psi0=Psi0, rho0=rho0, aTau=aTau, bTau=bTau))

###################
## MCMC SETTINGS ##
###################

## Setting for the overall run
##
numReps    <- 20000000
thin       <- 10000
burninPerc <- 0.5

## Settings for storage
##
nGam_save <- 0
storeV    <- rep(TRUE, 3)

## Tuning parameters for specific updates
##
##  - those common to all models
mhProp_theta_var  <- 0.05
mhProp_Vg_var     <- c(0.05, 0.05, 0.05)
##
## - those specific to the Weibull specification of the baseline hazard functions
mhProp_alphag_var <- c(0.01, 0.01, 0.01)
##
## - those specific to the PEM specification of the baseline hazard functions
Cg        <- c(0.2, 0.2, 0.2)
delPertg  <- c(0.5, 0.5, 0.5)
rj.scheme <- 1
Kg_max    <- c(50, 50, 50)
sg_max    <- c(max(data$rtime[data$recur == 1]),
               max(data$dtime[data$recur == 0 & data$death == 1]),
               max(data$dtime[data$recur == 1 & data$death == 1]))

time_lambda1 <- seq(1, sg_max[1], 1)
time_lambda2 <- seq(1, sg_max[2], 1)
time_lambda3 <- seq(1, sg_max[3], 1)

##
mcmc.WB  <- list(run=list(numReps=numReps, thin=thin, burninPerc=burninPerc),
                 storage=list(nGam_save=nGam_save, storeV=storeV),
                 tuning=list(mhProp_theta_var=mhProp_theta_var,
                             mhProp_Vg_var=mhProp_Vg_var, mhProp_alphag_var=mhProp_alphag_var))

##
mcmc.PEM <- list(run=list(numReps=numReps, thin=thin, burninPerc=burninPerc),
                 storage=list(nGam_save=nGam_save, storeV=storeV),
                 tuning=list(mhProp_theta_var=mhProp_theta_var,
                             mhProp_Vg_var=mhProp_Vg_var, Cg=Cg, delPertg=delPertg,
                             rj.scheme=rj.scheme, Kg_max=Kg_max,
                             time_lambda1=time_lambda1, time_lambda2=time_lambda2,
                             time_lambda3=time_lambda3))

#####################
## Starting Values ##
#####################
```

```
##
Sigma_V <- diag(0.1, 3)
Sigma_V[1,2] <- Sigma_V[2,1] <- -0.05
Sigma_V[1,3] <- Sigma_V[3,1] <- -0.06
Sigma_V[2,3] <- Sigma_V[3,2] <- 0.07


#############
## Data analysis: PEM-DPM ##
#PEM: non-parametric mixture of piecewise exponential models
#DPM: non-parametric Dirichlet process mixture of multivariate normals
#############

##
myModel <- c("Markov", "PEM")
myPath  <- "Output/02-Results-PEM/"

startValues      <- initiate.startValues_HReg(form, data, model=myModel, nChain=2)

##
fit_PEM <- BayesID_HReg(form, data, id=NULL, model=myModel,
                        hyperParams, startValues, mcmc.PEM, path=myPath)

fit_PEM
summ.fit_PEM <- summary(fit_PEM); names(summ.fit_PEM)
summ.fit_PEM
```

S4.2 The AFT additive-frailty model

```
library(SemiCompRisks)
library(Formula)
library(readxl)

#read data
data <- as.data.frame(read_excel("breast.xlsx"))
data<-data[data$nodes>0,]



#define variables
data$age_10<-data$age/10
data$size20_50<-ifelse(data$size=="20-50",1,0)
data$size50<-ifelse(data$size==">50",1,0)
data$log_nodes<-log(data$nodes)
data$log_pgr<-log(1+data$pgr)
data$log_er<-log(1+data$er)
data$grade3<-ifelse(data$grade==3,1,0)
data$age_since_relapse_10<-data$age_10+data$rtime/10

#define the times used in the format of package SemiCompRisks (in years)
data$y1L<-data$y1U<-data$rtime/365.25
data$y1U[which(data$recur == 0)] <- Inf
data$y2L<-data$y2U<-data$dtime/365.25
data$y2U[which(data$death == 0)] <- Inf
data$LT <- rep(0, dim(data)[1])


#define the formula that includes all transitions
form <- Formula(LT | y1L + y1U | y2L + y2U  ~
    age_10+meno+size20_50+size50+log_nodes+log_er+log_pgr+hormon+chemo+grade3 |
```

```
    age_10+meno+size20_50+size50+log_nodes+log_er+log_pgr+hormon+chemo+grade3 |
    age_since_relapse_10+meno+size20_50+size50+log_nodes+log_er+log_pgr+hormon+chemo+grade3)

#####################
## Define the Hyperparameters
#####################

## Subject-specific random effects variance component
##
theta.ab <- c(0.5, 0.05)

## log-Normal model
##
LN.ab1 <- c(0.3, 0.3)
LN.ab2 <- c(0.3, 0.3)
LN.ab3 <- c(0.3, 0.3)

## DPM model
##
DPM.mu1 <- log(12)
DPM.mu2 <- log(12)
DPM.mu3 <- log(12)

DPM.sigSq1 <- 100
DPM.sigSq2 <- 100
DPM.sigSq3 <- 100

DPM.ab1 <-  c(2, 1)
DPM.ab2 <-  c(2, 1)
DPM.ab3 <-  c(2, 1)

Tau.ab1 <- c(1.5, 0.0125)
Tau.ab2 <- c(1.5, 0.0125)
Tau.ab3 <- c(1.5, 0.0125)

##
hyperParams <- list(theta=theta.ab,
                    LN=list(LN.ab1=LN.ab1, LN.ab2=LN.ab2, LN.ab3=LN.ab3),
                    DPM=list(DPM.mu1=DPM.mu1, DPM.mu2=DPM.mu2,
                    DPM.mu3=DPM.mu3,
                    DPM.sigSq1=DPM.sigSq1,
                    DPM.sigSq2=DPM.sigSq2,
                    DPM.sigSq3=DPM.sigSq3,
                    DPM.ab1=DPM.ab1,
                    DPM.ab2=DPM.ab2,
                    DPM.ab3=DPM.ab3,
                    Tau.ab1=Tau.ab1,
                    Tau.ab2=Tau.ab2,
                    Tau.ab3=Tau.ab3))

###################
## Define the MCMC SETTINGS ##
###################

## Setting for the overall run
##
numReps    <- 1000000
thin       <- 1000
burninPerc <- 0.5

## Setting for storage
```

```r
##
nGam_save <- 10
nY1_save <- 10
nY2_save <- 10
nY1.NA_save <- 10

## Tuning parameters for specific updates
##
##  - those common to all models
betag.prop.var        <- c(0.01,0.01,0.01)
mug.prop.var        <- c(0.1,0.1,0.1)
zetag.prop.var        <- c(0.1,0.1,0.1)
gamma.prop.var        <- 0.01

##
mcmcParams <- list(run=list(numReps=numReps, thin=thin, burninPerc=burninPerc),
    storage=list(nGam_save=nGam_save,
    nY1_save=nY1_save, nY2_save=nY2_save,
    nY1.NA_save=nY1.NA_save),
    tuning=list(betag.prop.var=betag.prop.var,
    mug.prop.var=mug.prop.var,
    zetag.prop.var=zetag.prop.var,
    gamma.prop.var=gamma.prop.var))

############
## Analysis
############

#########
## DPM (Semiparametric Dirichlet process mixture model)##
#########

##
myModel <- "DPM"
myPath  <- paste0("Output/02-Results-DPM/",numReps,"/")

#initiate start values
startValues      <- initiate.startValues_AFT(form, data, model=myModel, nChain=3)

#fit
fit_DPM <- BayesID_AFT(form, data, model=myModel, hyperParams,
                       startValues, mcmcParams, path=myPath)

#summary
summ.fit_DPM <- summary(fit_DPM); names(summ.fit_DPM)
summ.fit_DPM

write.csv(summ.fit_DPM$theta,file=paste0(myPath,r,"_","theta_DPM.csv"))
write.csv(summ.fit_DPM$coef,file=paste0(myPath,r,"_","coef_DPM.csv"))
write.csv(summ.fit_DPM$psrf,file=paste0(myPath,r,"_","psrf_DPM.csv"))
write.csv(summ.fit_DPM$h0,file=paste0(myPath,r,"_","h0_DPM.csv"))
write.csv(summ.fit_DPM$setup[6:11],file=paste0(myPath,r,"_","setup_DPM.csv"))
```

S5 Glossary of Notation

$\mathcal{S}$ - the set of states.

$\mathcal{A}$ - the set of absorbing states.

$Z(t)$ - the state occupied at time t, where t is the time since the origin of a process.

$\{Z(s), 0 \leq s\}$ - the multistate process.

$H(t) = \{Z(s), 0 \leq s < t\}$ - the history of the process up to (not including) time t.
T_k - the entry time to state k, useful for progressive processes.
$T_k^{(r)}$ - the rth entry time to state k.
$N_{kl}(t)$ - the number of direct $k \to l$ transitions during $[0,t]$.
$\lambda_{kl}(t|H(t))$ - the $k \to l$ intensity function given the history $H(t)$.
$\Lambda_{kl}(t|H(t))$ - the cumulative transition intensity.
$p_{kl}(s,t) = \Pr(Z(t) = l|Z(s) = k)$ - the transition probability of $k \to l$.
$p_k(t) = \Pr(Z(t) = k|Z(0) = 0)$ - the probability that state k is occupied at time t.

C_i^* - a random right-censoring time of individual i.
τ - a fixed administrative censoring time.
$C_i = \min(C_i^*, \tau)$
$Y_i(t) = I(t \leq C_i)$ - equals 1 if individual i is uncensored prior to time t and 0 otherwise.
$Y_{ik}(t) = I(Z_i(t) = k)$ - equals 1 if state k is occupied at time t by individual i.
$\overline{Y}_{ik}(t) = Y_i(t)Y_{ik}(t^-)$ - equals 1 if individual i may be observed to transit out of state k at time t.
$\overline{Y}_{\cdot k}(t) = \sum_{i=1}^n \overline{Y}_{ik}(t)$ - the total number of individuals at risk of a $k \to l$ transition at time t.
$N_{ikl}(t)$ - the total number of direct $k \to l$ transitions over $[0,t]$ of individual i.
$\Delta N_{ikl}(t) = N_{ikl}((t+\Delta t)^-) - N_{ikl}(t^-)$ - the number of events of individual i over $[t, t+\Delta t)$.
$dN_{ikl}(t) = \lim_{\Delta t \downarrow 0} \Delta N_{ikl}(t)$
$d\overline{N}_{ikl}(t) = \overline{Y}_{ik}(t)\, dN_{ikl}(t)$ - equals 1 if a $k \to l$ transition is recorded for process i at time t.
$\overline{N}_{ikl}(t) = \int_0^t \overline{Y}_{ik}(s)\, dN_{ikl}(s)$ - the total number of observed direct $k \to l$ transitions of individual i over the interval $[0,t]$.
$d\overline{N}_{\cdot kl}(t) = \sum_{i=1}^n \overline{Y}_{ik}(t) dN_{ikl}(t)$ - the total number of observed $k \to l$ transitions at time t.

$X_i(t)$ - a $p \times 1$ vector of covariates at time t of individual i.
$\{X_i(t), 0 \leq t\}$ - the covariates' path up to time t of individual i.
$\mathcal{H}_i(t) = \{Z_i(s), X_i(s), 0 \leq s < t\}$ - the extended version of $H_i(t)$, includes the history of the multistate process and the covariates' path up to time t.

$C_i^*(t) = I(C_i^* \leq t)$ - equals 1 if random censoring occurred by time t, and 0 otherwise.
$Y_i^\dagger(t) = I(Z_i(t) \notin \mathcal{A})$ - the occupied state at time t by individual i is a non-absorbing state.
$\overline{Y}_i(t) = Y_i(t)Y_i^\dagger(t^-)$ - equals 1 if individual i may be observed to transit out of any state k at time t, $k \notin \mathcal{A}$.
$\overline{N}_{ik}(t) = (\overline{N}_{ikl}, l \neq k, l = 1, \ldots, K)^\mathsf{T}$ - vector of the cumulative number of transitions from state k over $(0,t]$.
$\overline{N}_i(t) = (\overline{N}_{ik}^\mathsf{T}(t), k \notin \mathcal{A})^\mathsf{T}$ - vector of all counting processes of non-absorbing states.
$\Delta\overline{X}_i(t) = \overline{Y}_i(t+\Delta t)\{X_i((t+\Delta t)^-) - X_i(t^-)\}$ - the increment in the covariate vector over $[t, t+\Delta t)$.
$d\overline{X}_i(t) = \lim_{\Delta t \downarrow 0} \Delta X_i(t)$
$\overline{X}_i(t) = \int_0^t d\overline{X}_i(s)$
$\overline{\mathcal{H}}_i(t) = \{Y_i(s), \overline{N}_i(s), \overline{X}_i(s), 0 \leq s < t; Z_i(0), X_i(0)\}$
$0 = u_0 < u_1 < \cdots < u_R = \tau$ - a partition of $[0, \tau]$.
$\Delta\overline{X}_i(u_r) = \overline{Y}_i(u_r)(X_i(u_r) - X_i(u_{r-1}))$ - an increment in the covariate vector of individual i.
$\Delta\overline{N}_i(u_r)Y_i(u_r)(N_i(u_r^-) - N_i(u_{r-1}^-))$ - the number of transitions of individual i over the $[u_{r-1}, u_r)$.
$\overline{H}_i(u_r) = \{Y_i(u_s), \Delta\overline{N}_i(u_s), \Delta\overline{X}_i(u_s), s = 1, \ldots, r; Z_i(0), X_i(0)\}$

A_i - the recruitment time of individual i.
$Y_i^A(t) = I(A_i \leq t)$ - individual i is recruited before time t.
$\tilde{Y}_i(t) = Y_i^A(t)Y_i(t)Y_i^\dagger(t^-)$ - equals 1 if individual i may be observed to transit out of any state k at time t, $k \notin \mathcal{A}$.
$\tilde{Y}_{ik}(t) = Y_i^A(t)Y_i(t)I(Z_i(t^- = k)$ - the individual is under study and at risk of a transition out of state k at time t.

$d\tilde{N}_{ikl}(t) = \tilde{Y}_{ik}(t) dN_{ikl}(t)$

$\tilde{N}_{ik}(t) = (\tilde{N}_{ikl}, l \neq k, l = 1, \ldots, K)^{\mathsf{T}}$ - vector of the cumulative number of transitions from state k over $(0, t]$.

$\tilde{N}_i(t) = (\tilde{N}_{ik}^{\mathsf{T}}(t), k \notin \mathcal{A})^{\mathsf{T}}$ - vector of all counting processes of non-absorbing states.

$\tilde{\mathcal{H}}_i(t) = \{Y_i^A(s), Y_i(s), d\tilde{N}_i(s), 0 \leq s < t, X_i\}$

$A_i(s)$ - the number of assessments of individual i up to time s.

$dA_i(t) = A_i(t) - A_i(t^-)$ - equals 1 if an assessment occurred at time t, and 0 otherwise.

$d\overline{A}_i(t) = Y_i(t) A_i(t)$

$\overline{A}_i(t) = \int_0^t d\overline{A}_i(s)$

$\overline{\mathcal{H}}_i(t) = \{Y_i(s), \overline{A}_i(s), d\overline{N}_i(s), 0 \leq s < t, X_i\}$

$0 \leq a_{i0} < a_{i1} < \cdots < a_{im_i}$ - assessments times of individual i.

$\overline{\mathcal{H}}_i^{\circ}(t) = \{Y_i(s), \overline{A}_i(s), 0 \leq s < t, (Z_i(a_r), a_r), r = 0, 1, \ldots, \overline{A}_i(t^-), X_i\}$
